# From images to health insight: integrating MLLM, NLP, and objective Q-sorting of nursing-home built environment orientations

**DOI:** 10.3389/fmed.2025.1737721

**Published:** 2026-01-07

**Authors:** Si-Jie Li, Zi-Jie Zou, Xi Ye, Chunhua Lin

**Affiliations:** 1 Faculty of Humanities and Arts, Macau University of Science and Technology, Taipa, Macau SAR, China; 2 School of Art/Pearl River Film Academy, Jinan University, Guangzhou, China; 3 School of the Arts, Universiti Sains Malaysia, Penang, Malaysia

**Keywords:** nursing homes, building environment (BE), natural language processing, multimodal large language models (MLLM), Q methodology, healthy aging, long term care facilities

## Abstract

**Background:**

Population aging has intensified pressure on global healthcare and social security systems, driving a shift in care from treatment-oriented approaches toward functional maintenance and chronic disease rehabilitation. How to design and optimize the built environment of nursing homes to support the physical and mental health of older adults has become an important issue in health policy and architectural design. Existing research lacks comparable types of environmental orientations for nursing homes and an operational guidance framework for environmental design, leaving subjective decision-making unable to align with functional maintenance goals.

**Methods:**

This study constructed a “semantic-cognitive” hybrid framework. It treated nursing homes' self-selected built-environment images on eldercare portals as espoused environmental orientation signals, revealing their belief structures and value orientations in convalescent practice. We compiled 3,578 environmental images from 389 nursing homes; used multimodal large language models (MLLMs) to generate structured environmental audit texts; applied natural language processing (NLP) for vectorization, dimensionality reduction, and clustering to refine and standardize the Q statement set; constructed Q-sorting similarity matrices from semantic similarity; and performed factor analysis with rotation to obtain typified belief structures.

**Results:**

Q methodology identified a four-factor solution explaining 86% of the total variance. Four environmental orientation types were identified in chronic disease management settings—Interior-centric type (safe accessibility, low stimulation, uniform lighting); Layout-oriented type (continuous corridors, clear entrances, orderly walking); Landscape-centered type (shaded gardens, good greenery, encouraging outdoor stay and social interaction); and Rehabilitation-driven type (rehabilitation equipment in place, open space, normalized training).

**Conclusion:**

This study provides a comparable and testable research pathway, reveals the linkage pathways between different environmental orientations and health-support mechanisms, and offers clear targets for subsequent longitudinal and mixed-methods evaluations, design, and evidence-based healthy aging policy management, with important theoretical and managerial significance.

## 1 Introduction

From a global perspective, the steadily growing older population and the rising incidence of non-communicable diseases (NCDs) pose unprecedented challenges to national health and social protection systems (1). Declines in self-care capacity and heightened risks of functional deterioration are prompting eldercare and long-term care systems to shift from a “treatment-centered” supply logic toward a sustained pathway that emphasizes functional maintenance and health restoration (2, 3). Prior experience indicates that integrating the diverse services required by older adults with multimorbidity into a single system facilitates higher-quality integrated care (e.g., greater service efficiency, improved patient experience, and better clinical outcomes) (2, 4, 5). Consequently, against the backdrop of accelerated demographic change, shrinking household size, intergenerational living apart, and normalized labor mobility (6), long-term care (LTC) facilities—including nursing homes—have, for many families, shifted from a “last resort” to a “rationally optimal choice.”

A favorable residential environment has been repeatedly shown to be systematically associated with physical and mental health, and the dynamic interactions between older adults and their residential environments play a crucial role in health recovery and quality of life (7–10). For older adults with chronic diseases, the triggers of behavior change may also be related to physical and social environmental characteristics (11). As patient needs become increasingly complex, nursing homes bear multiple, overlapping functions—safe care, daily residence, social participation, and emotional support—thereby increasing the difficulty of institutional care (12–14). Therefore, for care institutions to truly support healthy aging, their built environments must be well-matched to diverse real-world needs (15). Creating supportive environments can not only improve the quality of life of older adults but also reduce caregiver burden and yield potential economic benefits (16).

Institutional environmental orientation, as externally presented, not only shapes perceptions among potential users and family members but also, in turn, influences facilities' resource allocation and improvement pathways for healthy aging (17, 18). Accordingly, governance in the health sector is increasingly required to employ transparent, comparable environmental representations to promote institutional quality management and improvement, enhancing resident experience and service efficiency (19, 20). Therefore, establishing a scalable, objective audit process to classify the environmental orientations of care institutions not only facilitates recognition and comparison in public choice, but also provides connectable evidence between policy instruments and institutional communication (21).

However, existing research on this topic still has the following gaps: First, prior studies have primarily focused on the effects of built-environment features on health recovery [e.g., (7, 22)], but have seldom delineated the environmental priorities and trade-off structures from the perspective of nursing facilities, making it difficult to align the belief structures of NCDs-focused convalescent care with public needs. Second, although Q-methodology has unique strengths in identifying diverse viewpoints and typological structures (23, 24), at the institutional management level, there remains no clear and stable approach for studying and comparing environmental orientation types. In management-oriented inquiries, subjective information-gathering modes such as interviews or surveys are prone to bias or strategic responding, which may limit the complete capture of real conditions (25, 26). In contrast, the large samples of health resources related to eldercare or nursing available on portal websites comprise public materials (texts, environmental images, etc.) that reflect the quality and positioning that institutions wish to present and constitute attitudes and beliefs grounded in factual experience (27), which aligns with the governance needs of an objective Q approach. Finally, policy and public-health contexts require transparent and auditable channels to support cross-institutional comparison and public choice, yet existing research seldom provides evidence that can translate across the chain of concept presentation—design practice—regulatory evaluation (28). Therefore, incorporating public cues into scholarly comparison and establishing unified statement standards to increase cross-sample evidence accumulation can, to some extent, facilitate multimodal linkages between design guidance and regulation.

In summary, this study aims to develop a data-driven, scalable, objective auditing workflow that identifies long-term care facilities' belief structures regarding the built environment in chronic-disease rehabilitation and care, reveals their value orientations in environmental provisioning and convalescence practice, and aligns information flows between policy and stakeholders. Based on signaling theory, this study uses the environmental images that nursing institutions voluntarily display on online information platforms as orientation signals of their value priorities and care philosophy. These signals demonstrate how the institutions convey their prioritization of the living environment in elderly rehabilitation and reduce information asymmetry between institutions and users (29, 30). Compared with modifiable advertising text, images are widely used to extract spatial elements and service provision contexts; they serve as communication carriers and observable cue sets for the public and regulators and exhibit quasi-objective expressive properties in cross-institutional comparisons (31, 32). Specifically, the study has three sub-objectives: (1) to leverage large volumes of facility-released images and translate them, under a unified auditing standard, into objective and measurable descriptions of environmental features; (2) to quantify each facility's audited text in a way that preserves clinical semantics while enabling cross-institutional comparability; and (3) to construct corresponding institutional environmental profiles and identify orientation types, and—by distilling distinguishing and consensus statements—to provide actionable evidence for environmental design, clinical operations, and policy management that supports public choice and targeted optimization.

To this end, this study builds a hybrid model that couples a multimodal large language model (MLLM), natural language processing (NLP), and objective Q-methodology to systematically explore the above relationships. First, drawing on prior literature, we establish core built-environment dimensions aligned with healthy aging and NCD rehabilitation, and we scrape large volumes of environmental images as “conceptual cues” of convalescent care. Second, to reduce researcher subjectivity and enhance transparency of the evidence chain, we use the MLLM to produce consistent, repeatable, criterion-based descriptions of each facility's images (e.g., natural light/illumination, space layout/privacy, safety/assistive features, greenery/natural elements). From large numbers of images, spatial semantics and care-context information are automatically extracted, translating visual environments into analyzable audit text. Compared with manual auditing, the MLLM is better suited for handling large-scale standardized text outputs (33). Then, using NLP, we construct comparable facility-level representations by vectorizing and clustering these semantic texts to form a data-constrained statement set that serves as the objective structural basis for subsequent Q-sorts and factor analysis. Based on a semantic-similarity model, we compute a score matrix for each facility across statements. Finally, we input the results into the Q-methodology framework to reveal the latent belief dimensions of nursing homes regarding the built environment. The findings translate differences in combinations of objective visual features into comparable profiles of long-term care facilities, providing data-driven theoretical and methodological references for spatial design in nursing homes, optimization of chronic-disease convalescent care strategies, and environmental intervention policies.

## 2 Built-environment features in nursing homes and health recovery

### 2.1 Associations between interior environment factors and health

With advancing age, people tend to spend more time indoors; interior environmental factors significantly affect older adults' physical and mental health and are indispensable for supporting healthy aging (34). The scope of this study primarily concerns modifiable built-environment features in interior settings, such as lighting, layout, accessibility design, and furniture.

Prior research has shown that general physical health is associated with ventilation/air quality and lighting (34). Lighting has become the most comprehensively studied interior factor, particularly with respect to its effects on sleep, mood, and daily functioning. Older adults in nursing homes may have fewer opportunities for exposure to natural light than community-dwelling older adults (35). Appropriate lighting design affects visual function and decision-making and may mitigate depressive and anxious tendencies (36–38). Accordingly, increasing daylighting through windows and optimizing lighting design to ensure a bright and comfortable visual environment are critical to promoting health. Existing studies have extended dynamic correlated color temperature schemes or illuminance recommendations to daily activities (39, 40). Low levels of ventilation/air quality are associated with elevated indoor CO_2_ concentrations and increased cardiovascular strain; indoor plants are a practical and low-cost strategy to improve air quality (41).

In layout-related studies, bathroom configuration and bedroom privacy most clearly demonstrate key roles in supporting older adults' health (34). Related factors include the completeness of amenities within functional spaces, spatial connectivity between functional zones, and the degree of control over personal rooms (35). Visibility, perceived openness, and accessibility of space are critical for older adults; adequate space facilitates the use of walkers and enhances social interaction, whereas overly narrow spaces may increase depressive symptoms and extreme affect among older adults with dementia (42). Broad sightlines and the ability to view natural scenery from indoors can also substantially enhance positive perceptions (43).

As a key enabler of mobility and daily functioning in older adults, accessibility design is increasingly integrated with multisensory visual strategies (44, 45). This involves not only installing infrastructure such as call buttons in living spaces (46) but also emphasizing furniture, door and window design, material and color schemes, and visual access to exterior landscapes. For example, warm, bright colors can enhance spatial awareness (47), and older adults with dementia are more likely to exhibit relaxed, calm, and pleasant affect in environments with soft color palettes (48). In addition, personalized door decorations can aid wayfinding memory among older adults with cognitive impairment (38); high color-contrast handrails against background walls, floors, and doors made of materials with low reflectance, and LED guide lights are also strategies to improve visibility and reduce fear of falling (36, 49). Prior research has shown that when wood coverage in activity rooms for older adults is about 30%, subjective responses are most positive, whereas 90% coverage produces a significant calming effect (50). In recent healthcare settings, the discussion of furniture has expanded to deeper health dimensions—such as cognitive performance, loneliness, and satisfaction (36, 38)—reflecting a shift in furniture design from purely functional considerations toward affective and symbolic roles. For instance, open kitchens or dining spaces may encourage residents to gather and engage in daily activities, providing a supportive social environment (51).

### 2.2 Associations between outdoor environment factors and health

Research over recent decades has demonstrated that outdoor environments can serve as resources for recovery and rehabilitation, leading to the establishment of gardens in healthcare settings (52). The health benefits of outdoor activities for older adults have been extensively discussed, with their psychological, cognitive, emotional, mobility, and physical function linked to diverse outdoor spaces (53–55), including improved mood, sleep, wellbeing, and quality of life (56–58). Despite the well-established positive impact of outdoor natural landscapes on health and wellbeing, gaps remain in understanding their effects on individuals with dementia residing in long-term care (LTC) facilities (59).

For residents with dementia living in nursing homes, the value of outdoor natural landscapes within the healthcare system is associated with characteristics of dementia-friendly environments, including orientation, accessibility, socialization, meaningful activities (meaningful engagement), sensory stimulation, reminiscence, safety, and sustainability, in order to meet their needs and maximize the therapeutic potential of outdoor natural landscapes (59). Previous studies have identified the following drivers of outdoor space utilization in nursing homes: garden planting and architectural features, safety concerns and staffing, adequate shelter, the design of main-building entrances, and social activities (56). Therefore, providing residents with opportunities for plant cultivation in gardens, equipping spaces with interactive facilities, weather-protected seating, and accessible entrances, and organizing social activities are crucial for facilitating their engagement with outdoor environments.

Expansive, aesthetically pleasing outdoor landscapes and greenery have restorative effects on older adults, helping them maintain a positive mood (59). Biophilic experiences in walking environments—such as green spaces, vegetation, and natural visual simulations—provide positive sensory stimulation, significantly reducing participants' stress and anxiety and offering measurable therapeutic benefits (55). Therefore, providing sufficient pathways and walkable surfaces is crucial to enable safe movement (56). Installing handrails in outdoor walking areas to assist residents with mobility and balance is recognized as enhancing a sense of safety (60). Furthermore, outdoor spaces and green spaces should offer ample opportunities for social interaction among residents, fostering engagement and strengthening a sense of belonging (61). Adequate seating that is easily accessible, comfortable, and not overly hard is necessary for residents to enjoy garden spaces (56). Heavy or locked doors and high thresholds or doorsteps present barriers to independent outdoor access for older adults, particularly those using walkers (62). Several studies also emphasize the need for adequate lighting in outdoor spaces, especially at night (56, 63). Some nursing-home residents desire views beyond garden boundaries to observe surrounding neighborhoods or businesses, fostering a connection to life outside the facility (64).

Although assisted living environments primarily focus on physical safety and visual supports, integrating cultural and psychological design elements is also crucial (61). The cultural characteristics of nursing homes, and their connections with the broader society, support nursing-home residents' continued sense of belonging and engagement in the local community (65). This specific culture arises not only from the words and actions of fellow residents and staff but is also manifested in details such as decor and furnishings. The provision of personalized care enhances residents' physical safety and emotional wellbeing in later life (34). Specific dimensions and indicators used for MLLM description are shown in Table 1.

**Table 1:** Descriptions of potential dimensions and associated indicators.

**Dimensions**	**Indicators**	**Reference**
Indoor dimension	1.Daylighting and lighting conditions (window orientation and area; number and color/temperature of luminaires; blackout/shading curtains).	(36, 37, 45)
	2.Ventilation and indoor air quality (e.g., operable doors/windows; mechanical ventilation/air-purification devices).	(36, 45)
	3.Spatial layout and privacy (open-plan vs. compartmentalized functional zones; corridor length; extent of visual exposure/sightlines).	(34, 42, 45, 103, 104)
	4.Safety and assistive features (e.g., bathroom grab bars; corridor lighting; handrail-wall luminance contrast; wayfinding signage; nurse-call buttons).	(45, 105)
	5.Spatial capacity (e.g., overall spaciousness; wheelchair accessibility and maneuvering clearance).	(43, 45, 106)
	6.Comfortable and flexible furniture (e.g., relative placement of beds to windows/desks; social seating configurations).	(34, 45, 49)
	7.Environmental aesthetics and art elements (e.g., decorative objects and style; indoor plants; proportion of timber; material reflectance; color palette and saturation).	(45, 107)
Outdoor dimension	1.Greening and natural elements (e.g., vegetation coverage; species richness and health; water features).	(56, 59, 108)
	2.Accessible design and facilities (e.g., entrances and site boundaries; fencing; handrails; sidewalk/path width; outdoor furniture; color contrast).	(59, 109)
	3.Main-building design (e.g., façade articulation/complexity; architectural style; materials; color scheme; visual interest).	(56, 110)
	4.Entrance forecourt and seating (e.g., provision of seating in the entrance plaza; steps; landscape ornaments; scale/proportions).	(56, 61, 111, 112)
	5.Paths and Surface conditions (e.g., walkway width; paving materials; diversity of route types).	(56, 61)
	6.Shade and shelter (e.g., shaded/rain pavilions; terraces; overhead canopies).	(52, 56)
	7.Visibility and night-time lighting (e.g., pedestrian-path and façade lighting provision).	(61, 113)
	8.Proximity and activity intensity (e.g., connectivity to commercial streets; openness; streetscape vibrancy; access to public services).	(56, 114)
	9.Environmental quality (e.g., cleanliness and maintenance; condition of facilities; orderliness).	(115, 116)
	10.Social opportunities (e.g., activity/interaction amenities; furniture for gathering; family-visit and group-activity facilities).	(56, 60, 61)
Free dimension	Identify and add any salient environmental attributes not covered above—for example, cultural symbols, pet-friendly amenities, digital devices/displays, or specialized activity rooms.	(60, 117, 118)

## 3 Materials and methods

### 3.1 Overview

**Figure 1:**
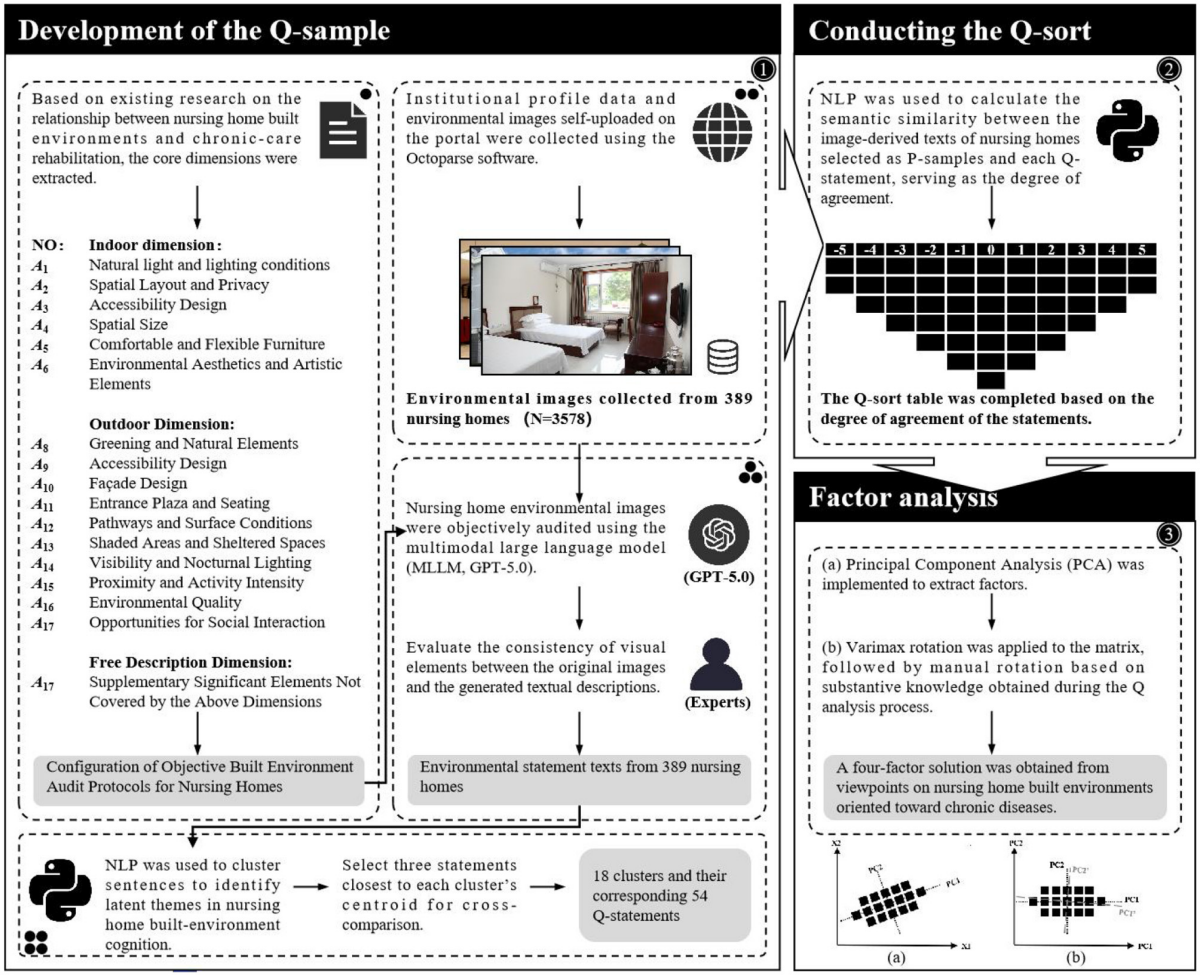
Research process and overall framework.

This study aims to identify the belief structures held by nursing homes regarding the built environment for chronic disease rehabilitation care, thereby revealing their value orientations in rehabilitation practices and environmental design. To this end, a hybrid “semantic-cognitive” framework integrating multimodal language models (MLLM), natural language processing (NLP), and Q-methodology was constructed to systematically explore these relationships (see Figure 1).

Q methodology is regarded as a research approach that investigates individual perceptions via the application of inverted factor analysis (66). While it can characterize holistic responses and aggregate them into stable configurations of viewpoints (67), its primary limitations lie in preparatory information gathering and Q-statement construction: the selection of contextual materials and wording is influenced by researchers' choices, making it difficult to fully eliminate the interaction between the subjectivities of the observer and the observed (67). Therefore, given the high accessibility of information on web portals, the environmental images and supporting materials that institutions select and publish on these portals provide explicit signals of their spatial orientations and care philosophies. Compared with template-based, highly stylized texts, images that directly present spatial elements and service contexts better reflect underlying beliefs. These beliefs can be viewed as the externalization of practical knowledge—transformed from accumulated experience—by institutional managers, frontline caregivers, designers, and other stakeholders through long-term work with older adults, information that has yet to be systematically explored.

To limit discretion and enhance transparency in the chain of evidence, this study employs MLLM and NLP as upfront preprocessing to provide data-constrained sources for the statements. First, MLLM automatically extracts spatial semantics and care-context information from images scraped from web portals, converting visual representations of the built environment into analyzable textual descriptions. Subsequently, NLP vectorizes and clusters these semantic texts to construct a data-constrained Q-statement set, and a semantic similarity model computes an institution-by-statement scoring matrix that serves as the objective structural basis for subsequent Q-sorting and factor analysis. The methodological process includes defining and constructing the concourse, developing the Q-set, selecting the P-set, and conducting Q-sorting, analysis, and interpretation (66, 67). The following subsections detail the Q-methodological steps of this study.

### 3.2 Development of the Q-sample

**Figure 2:**
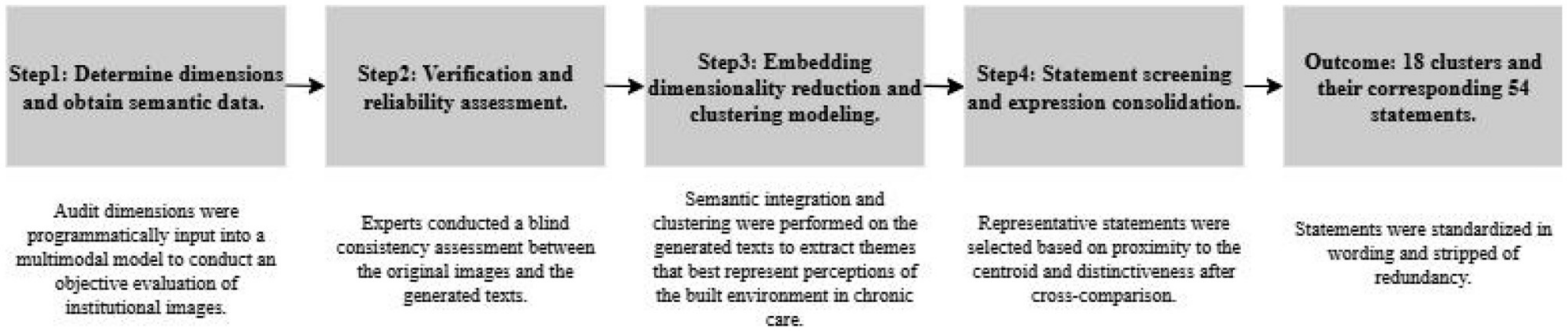
Procedure for constructing the Q sample.

The concourse is the entirety of discourse concerning a specific research question (68) and is constructed by gathering statements that represent a broad range of subjective perspectives on that issue. Subsequently, a final set of statements—the Q sample—must be selected. It must “broadly represent the opinion domain” and should “demonstrate good coverage relevant to the research question.” The collection process includes both structured and unstructured methods (69). Q methodology does not define a specific procedure for generating statements. In practice, Q sets are created based on scientific literature, policy documents, mass media, structured interviews, and informal discussions with experts (70). Following the unstructured approach, this section derives “data-constrained” Q-sample statements by integrating MLLM with NLP, with the process steps illustrated in Figure 2.

Step 1: Identify dimensions and acquire semantic data. The concourse is constructed based on existing research on the relationship between the built environment of nursing homes and chronic disease rehabilitation, from which core dimensions highly relevant to the research theme are distilled, specifically including: for indoor environments—“Daylighting and Lighting conditions,” “Ventilation and Air Quality,” “Spatial Layout and Privacy,” and “Safety and Assistive features”; for outdoor environments—“Greening and Natural elements,” “Paths and Surface conditions,” “Main-building design,” and “Social Opportunities.” In addition, a “free dimension” is set to capture unspecified features (see Supplementary materials for detailed instruction configuration). These dimensions are used to instruct a multimodal language model (MLLM) to objectively audit environmental images self-selected and uploaded by institutions to nursing-home web portals, thereby generating descriptive text of the images. MLLMs have been shown to achieve high accuracy and stability in the semantic understanding of images and the recognition of scene elements. This study employed GPT-5.0 (71, 72), the latest iteration of such models.

Step 2: Verification and Reliability Assessment. To verify the usability and consistency of the model-generated text, this study randomly selected ~10% of the samples (358 images in total). Two experts with backgrounds in environmental design or elderly care assessed the consistency of elements between the original images and the generated text. The assessment results showed that the two experts classified 95.35 and 94.69% of the generated text as fully usable and 1.96 and 3.07% as partially usable, respectively. The proportion of unusable text was 2.52%. The kappa coefficient calculated based on the above classification results was 0.822 (95% CI: 0.658–0.945, *P* < 0.001). These results support a high level of consistency between assessors and demonstrate that the GPT output has good reliability in key element identification and semantic integrity.

**Figure 3:**
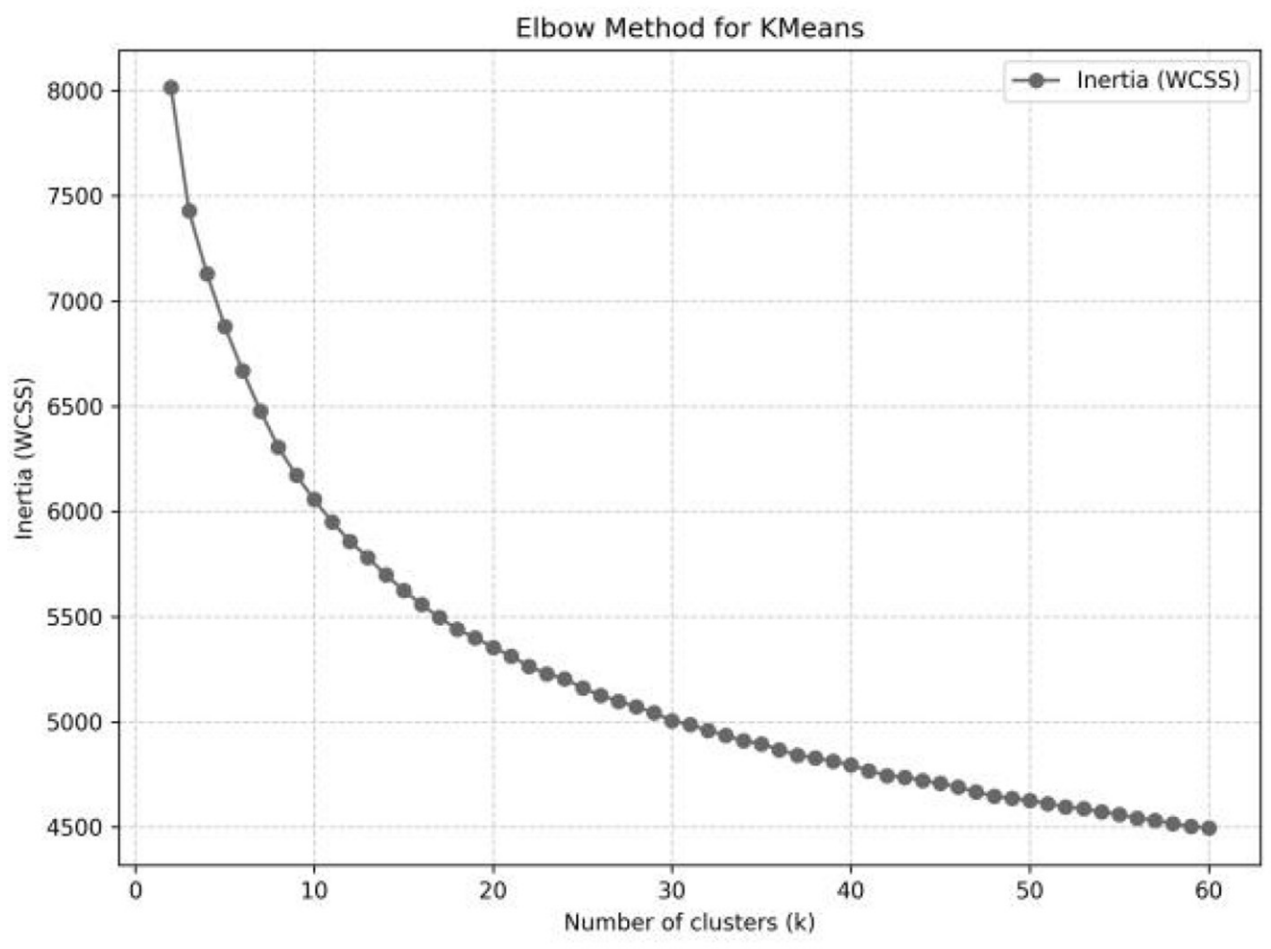
Elbow plot.

Step 3: Embedding, Dimensionality Reduction, and Cluster Specification. These qualitative texts extensively cover nursing homes' statements regarding perceptions of the built environment for chronic disease rehabilitation. Information extraction in this study is achieved through inductive analysis based on natural language processing (NLP). After sentence segmentation and normalization, semantic embeddings are generated with a multilingual SBERT model, followed by principal component analysis (PCA) for dimensionality reduction to mitigate noise and redundancy. K-means clustering is then applied to the semantic vector matrix to identify latent thematic structures. To determine the number of clusters *K*, an elbow curve was computed over a grid with *K* ∈*[2,60]* to delimit the candidate range; as shown in Figure 3, the curve exhibits diminishing marginal improvements for *K* ∈*[10,20]*, indicating the candidate interval. To further refine the choice of *K*, the silhouette score and the Davies-Bouldin index (DBI) were jointly used for validity assessment, where larger silhouette values indicate greater within-cluster compactness and better between-cluster separation, and smaller DBI values are preferred (73). Results showed that *K* = *4* achieved the best overall ranking (Silhouette = 0.097, DBI = 2.571). All computations were performed in PyCharm 2023.2.3.

Step 4: Statement Screening and Refinement. Based on the clustering results, the research team first selected three statements from each partition that were closest to the centroid and had a certain degree of discriminative power, resulting in 42 representative statements. Several near-optimal clustering solutions were then cross-referenced to check for any semantic categories marginalized by the optimal solution. Next, two researchers reviewed the initially selected statements, eliminating entries that were highly repetitive within the same or different partitions, covered multiple environmental dimensions, or were vaguely worded, making it difficult to maintain a consistent understanding. Through discussion, statements that both summarized the core meaning of each partition and were distinguishable were retained, ultimately determining 18 partitions and their corresponding 54 statements. Based on this, wording was standardized and redundancy was eliminated. Table 2 provides an example statement for each category. A complete list of statements and categories can be found in Supplementary Table S1.

**Table 2:** Q sample with example statements.

**Statement category**	**Number**	**Content of Q set**
Outdoor environment and activity accessibility (*C*_1_)	3	e.g., The environment is well-maintained and clean, the facilities are intact and orderly, the building is integrated with greenery, and the outdoor space is open, supporting surrounding activities and circulation.
Interior color scheme and decoration (*C*_2_)	3	e.g., The space features a fresh and warm color palette, with decorative artwork and bedding, meets residential needs, and is kept orderly.
Open-plan living area (*C*_3_)	3	e.g., The layout is open with a wide visual field, it is furnished with mahjong tables, seating, and sofas, the space is spacious and wheelchair-accessible, and it is suitable for group leisure and recreation.
Bathroom configuration (*C*_4_)	3	e.g., The restroom is finished with light-colored tiles, it is equipped with a countertop sink and mirror, a toilet with grab bars on both sides, and a shower area with a shower head, grab bars, and a shower chair.
Site greenery (*C*_5_)	3	e.g., The outdoor environment features abundant greenery and natural elements, trees and shrubs are well-maintained, and vegetation coverage is high and shows healthy growth.
Care bed arrangement (*C*_6_)	3	e.g., The dedicated functional area is furnished with a nursing bed and a bedside cabinet, bedding is complete, and daily-use items such as a fan are placed beside the bed.
Natural lighting and window arrangement (*C*_7_)	3	e.g., The interior features large windows with venetian blinds, allowing natural light in.
Lighting system (*C*_8_)	3	e.g., Ceiling-mounted fixtures, recessed downlights, and wall-mounted indirect lighting are provided.
Surface paving (*C*_9_)	3	e.g., The ground surface is paved with asphalt, which is even and easy to traverse.
Surface decoration and wayfinding signage (*C*_10_)	3	e.g., Decorative artwork and informational signage are affixed to the walls.
Building facade (*C*_11_)	3	e.g., The main exterior facade of the building is in light tones, the decorative style is simple, and the structure is multi-story.
Ventilation and air-conditioning system (*C*_12_)	3	e.g., Ventilation is achieved through operable external windows together with air conditioning.
Spatial scale and wheelchair circulation (*C*_13_)	3	e.g., The space is wide, and wheelchair circulation is possible.
Public-area seating and reception desk arrangement (*C*_14)_	3	e.g., Sets of tables and chairs are arranged in the common area, forming a social seating layout.
Entry roadway and pedestrian interface (*C*_15_)	3	e.g., A vehicular lane with an asphalt surface is provided on one side of the entrance, which forms a boundary with the adjacent pedestrian walkway.
Entrance structure (*C*_16_)	3	e.g., At the corner, an arched entrance and a gated wall opening are provided, and the entrance includes fencing and a header sign.
Rehabilitation equipment (*C*_17_)	3	e.g., The layout is open with a wide visual field, multiple sets of rehabilitation and exercise equipment and suspended training devices are provided.
Shaded outdoor circulation and resting nodes (*C*_18_)	3	e.g., An outdoor shaded corridor with a transparent roof, using a metal frame and translucent materials, is provided, with a tiled pedestrian walkway beneath it, flowerbeds and a variety of greenery including trees and shrubs alongside, the environment is orderly, and the corridor provides outdoor circulation and a place to rest.

### 3.3 Selection of the P-set

The P set refers to respondents who are as heterogeneous as possible in Q sorting and are theoretically relevant to the research topic, and they are also called Q participants. A qualified study requires 25–75 participants (24). This study uses nursing homes as the P sample unit. Data on established nursing homes in mainland China were collected through portals such as “Nursing Home Network” (https://www.yanglao.com.cn/). The collected data include basic information about the institutions and environmental images uploaded by the institutions themselves to the website. These environmental images are considered as explicit representations of value orientations and spatial concepts, and the set constitutes the overall statement. Therefore, the Q sorting in this study describes the environmental orientation at the organizational level, rather than the daily experience of residents or staff. The research design using institutions as the P set aims to classify and benchmark the types of nursing home environmental orientations at the institutional level for service governance and public communication scenarios, rather than as a direct auditing tool for daily environmental quality.

Using “nursing homes” as the search keyword, a total of 765 institutions of this type were found on the website. Subsequently, the open-source online data collection tool “Octopus” was used to systematically crawl the website data. After data collection, this study adopted a two-step data cleaning strategy that combined code screening and manual review. The script first completed duplicate detection and quality and relevance screening, and then manual sampling and correction were carried out according to the preset specifications to ensure the validity and consistency of the samples. After cleaning and removing invalid data, 389 valid sample institutions were retained, corresponding to 3,578 images. Basic information about these institutions is shown in Table 3. The P-set comes from 78 cities, with a wide geographical distribution of the participants and diverse economic regions. The included institutions also cover different types and sizes, thus meeting the heterogeneity requirements for sample selection.

**Table 3:** Statistical characteristics of the sample.

**Statistical indicator**	**Categorical indicator**	**Frequency**	**Percentage**
Type of institution	Publicly run	29	7.46%
	Privately run	299	76.86%
	Publicly built and privately operated	61	15.68%
Range of service fees	0–1,500	3	0.77%
	1,501–2,000	6	1.54%
	2,001–2,500	17	4.37%
	2,501–3,000	30	7.71%
	Above 3,000	343	88.17%
Bed capacity	0–99	69	17.74%
	100–499	246	63.24%
	Above 500	74	19.02%

### 3.4 Conducting the Q-sort

**Figure 4:**
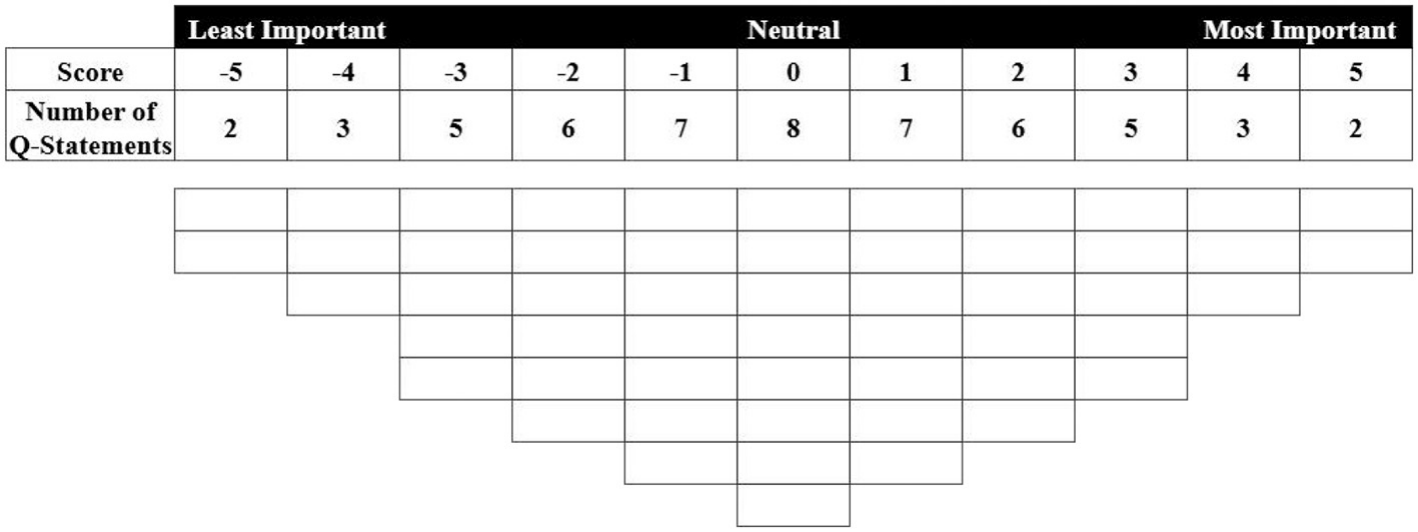
Q-sort grid.

Q sorting involves individual participants recording their subjective judgments of Q statements on a Q-sort grid. Unlike previous related studies, this research does not employ the traditional approach of collecting perspectives through expert or public self-reports. Instead, it calculates the semantic similarity between the image-derived texts of nursing homes selected as the P sample and each Q statement to derive their levels of agreement across statements. Statements are then assigned to the Q-sort grid based on these agreement levels, ranging from “least important” (−5) to “neutral” (0) to “most important” (+5). As shown in Figure 4, the Q-sort employs a fixed quasi-normal distribution, allocating scores across 11 levels. Following this process, each selected individual is represented by a Q-sort (74).

### 3.5 Factor analysis and interpretation

In Q methodology, factor analysis is an iterative process in which researchers identify and evaluate multiple factor solutions to find those interpretable as meaningful perspectives (68). However, identifying significant factors requires more than statistical measures; it also entails determining theoretical insights and qualitative considerations consistent with the Q design and dataset (75, 76). Data analysis was conducted using the embedded statistical functions of the online Ken-Q Analysis v2.01 web application (77). First, by-person correlations were computed to construct a correlation matrix; high pairwise correlations indicate overlapping organizational understandings of chronic disease-oriented built-environment practices, with each shared-meaning cluster represented by a distinct factor. Second, principal component analysis (PCA) was implemented on the correlation matrix for factor extraction, aiming to reduce dimensionality while maximizing explained variance (24). The matrix was then rotated (using Varimax as the baseline, with small-angle manual rotation when necessary) to achieve optimal loadings of Q sorts on the factors, thereby enhancing interpretability. Varimax rotation uses statistical criteria to maximize the total variance explained by the factors. Nevertheless, in Q methodology the optimal mathematical solution is not always the most meaningful one—i.e., the solution that best explains and illuminates diverse perspectives. Accordingly, it is recommended to apply Varimax first, followed by manual rotation informed by substantive knowledge obtained during the Q analysis (68). The rotated factor loadings indicate how well each individual/Q-sort aligns with each perspective, while minimizing the number of perspectives with high loadings for the same individual. Subsequently, scores for each statement within each factor are calculated to interpret meanings based on the statements most relevant to each perspective (74). Finally, the perspective framework is refined and clarified based on the *z*-scores for each factor and the resulting factor arrays. These *z*-scores and factor arrays reveal the statements ranked highest and lowest within each framework, enabling the naming and interpretation of each perspective. It is important to emphasize that the framework does not represent any single individual's opinion but rather an idealized position collectively presented by individuals representative of that factor (70). The aim is to synthesize dispersed individual rankings into a comparable shared discourse of action, thereby identifying differences and consensus across groups. On this basis, a typology of viewpoints can be constructed, systematically characterizing core insights into chronic disease-oriented built-environment practices according to the statements occupying high and low positions within each type.

## 4 Results and discussions

### 4.1 Factor analysis

**Figure 5:**
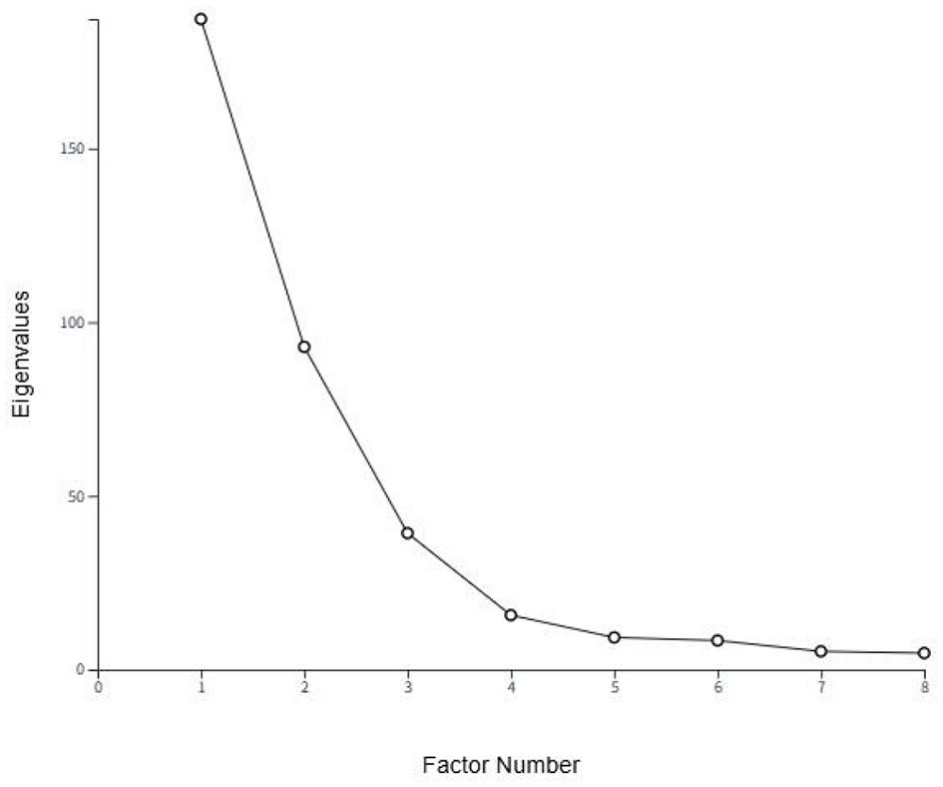
Scree plot.

The specific factor extraction procedure followed previous research (78), employing PCA to extract and examine one- to eight-factor solutions. Among the candidate solutions, the Kaiser-Guttman criterion was first used to assist in identifying the optimal solution; this statistical standard requires eigenvalues exceeding 1.0 as the threshold for factor inclusion, and each factor must have at least two Q sorts to achieve significant loading (66). The significance threshold (*P* < 0.01) was determined by dividing 2.58 by the square root of the total number of statements in the Q set (66). With 54 statements in this study, this yielded a threshold of 2.58/√54, ~0.351. Thus, only Q sorts with absolute loadings exceeding 0.35 on a given factor were considered statistically significant. According to the embedded functionality in Ken-Q Analysis v2.01, a Q sort must account for the majority of its communality on the target factor to be considered a defining Q sort for that factor. In addition, the inflection point on the screen plot in Figure 5 was jointly considered. Factor 4 represents a clear inflection point; beyond it, factor eigenvalues decline gradually and contribute little to the cumulative percentage. Applying these screening rules yielded a four-factor solution explaining 86% of the variance in the data (see Table 4).

**Table 4:** Eigenvalues of the unrotated factor matrix.

**Metrics**	**Factor 1**	**Factor 2**	**Factor 3**	**Factor 4**	**Factor 5**	**Factor 6**	**Factor 7**	**Factor 8**
Eigenvalues	187.302	92.909	39.265	15.6638	9.240	8.362	5.242	4.747
Explained variance	48	24	10	4	2	2	1	1
Cumulative explained variance	48	72	82	86	88	90	91	92

### 4.2 Factor rotation

Varimax rotation was evaluated through a preliminary interpretation of the four-factor solution, examining all Q sorts exhibiting significant loadings on one or more factors. Qualitative data subsequently revealed that three Q sorts—Q sorts 4, 317, and 375—with significant cross-loadings on two factors (borderline cases) actually aligned more closely with Factor 4. Therefore, following the rotation procedure outlined by Watts and Stenner (78), the research team performed a manual adjustment: rotating Factors 1 and 4 counterclockwise by two degrees. This increased the number of defining Q sorts in Factor 4 to five. Beyond these three Q sorts, the manual adjustment had no further impact on the factor structure. Thus, in the final four-factor solution, all 389 Q sorts loaded significantly on one or more factors, with 360 being defining Q sorts—those loading significantly on a factor and accounting for the majority share of their communality. Factor 1 had the highest number of defining Q sorts (180), followed by Factor 2 (135) and Factor 3 (40) (see Table 5).

**Table 5:** Factor characteristics.

**Characteristics**	**Factor 1**	**Factor 2**	**Factor 3**	**Factor 4**
No of defining variables	180	135	40	5
Avg Rel Coef	0.8	0.8	0.8	0.8
Composite reliability	0.999	0.998	0.994	0.952
S E of factor *Z* scores	0.032	0.045	0.077	0.219

### 4.3 Factor interpretation

**Figure 6:**
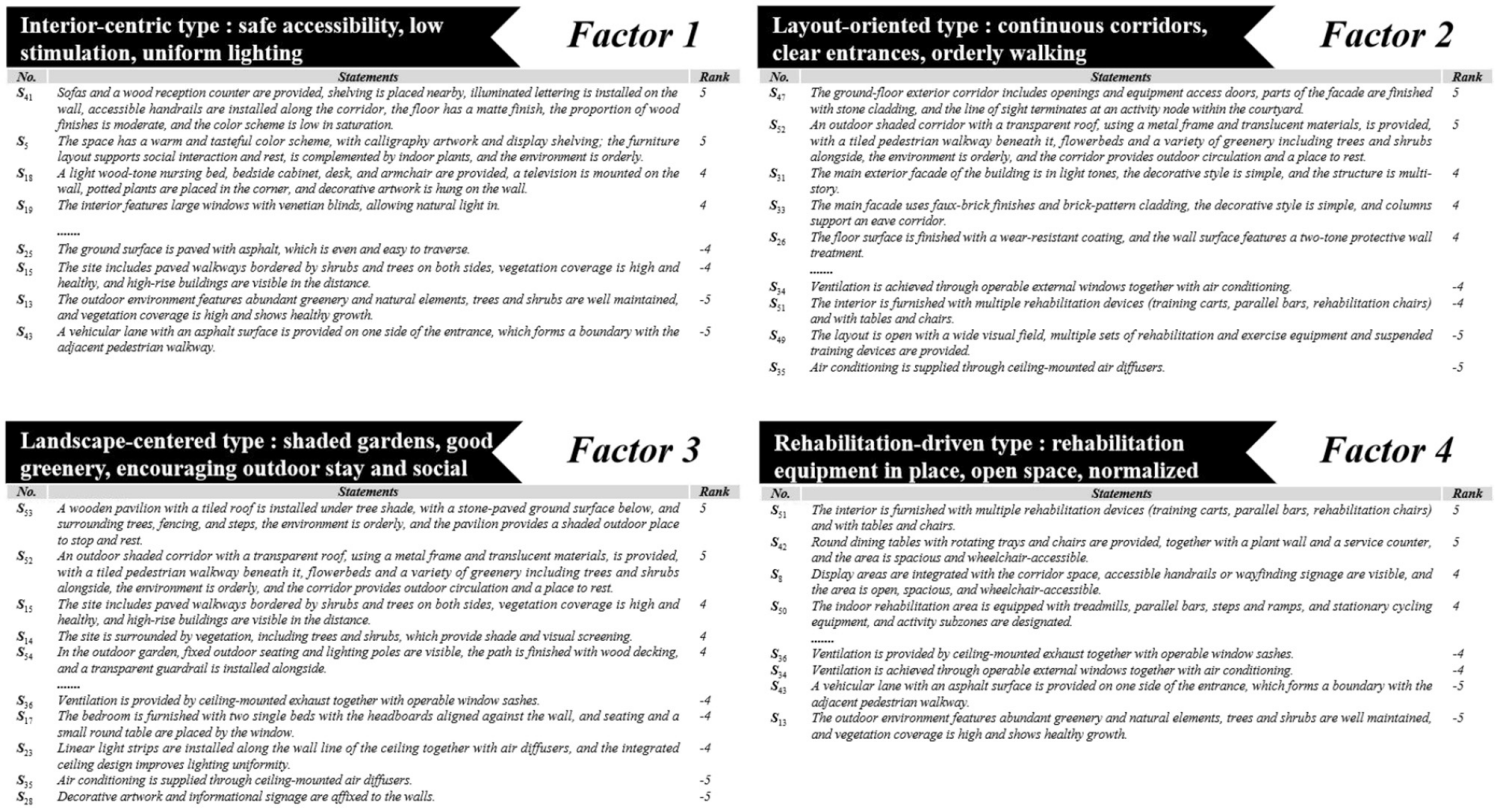
Four-factor results summary chart.

To deepen understanding of the four perspectives, this study interprets two types of statements: high- and low-ranked statements (the extreme columns of the factor array, per this study's forced distribution of ±4/±5), which delineate what each perspective most emphasizes and most opposes regarding chronic disease-oriented built-environment practices; and distinguishing statements, which identify items ranked significantly higher or lower than in other perspectives, thereby pinpointing key divergences (see Figure 6). Complete factor scores and significant factor loadings for each statement are presented in Supplementary Tables S2, S3.

#### 4.3.1 Interior-centric type: safe accessibility, low stimulation, uniform lighting

This perspective accounts for the largest share of variance and represents the prevailing view within care institutions. The high-ranking statements indicate that institutions within this factor group prioritize facilities centered on indoor risk control and accessibility as their core value orientation, exhibiting a consistent internal structure in their preferences. As shown in Table 6 (results for other factors are in Supplementary Tables S4–S6), the highest-scoring and most distinguishing items are concentrated on low-reflectance and low-saturation materials, structured wayfinding, and continuous handrail systems (*S*_41_ = 5; *S*_5_ = 5), with layered lighting and adjustable natural light forming the foundational environment (*S*_19_ = 4; *S*_21_ = 4; *S*_20_ = 3). This set of features points to a common mechanism: improving the predictability of visual cues by reducing glare and abrupt visual-contrast changes. Furthermore, continuous handrails and clear signage reduce cognitive load for orientation and walking (79), while uniform illuminance and accessible functional points lower wayfinding difficulty and fall risk along high-frequency pathways such as bedroom-bathroom-corridor (80). At the same time, adequate and circadian-friendly indoor lighting is a critical condition for compensating for age- and dementia-related functional deficits (81). Residential units are organized around the bed and furnished with nursing beds, bedside tables, desks, and storage shelves to accommodate extended stays, light social interaction, and caregiving needs (*S*_15_ = 5; *S*_18_ = 4; *S*_17_ = 4). This further demonstrates that Factor 1 prioritizes accessible and easy-to-maintain everyday micro-settings, aligning with the high-frequency demands of chronic disease care.

**Table 6:** Overview of the defining statements (±4 and 5) and distinguishing statements (at *P* < 0.05) for factor 1.

**No**.	**Statements**	**Factor 1**	**Con/dist**	**Factor 2**	**Factor 3**	**Factor 4**
*S*_41_	Sofas and a wood reception counter are provided, shelving is placed nearby, illuminated lettering is installed on the wall, accessible handrails are installed along the corridor, the floor has a matte finish, the proportion of wood finishes is moderate, and the color scheme is low in saturation.	5	D^*^	3	2	4
*S*_5_	The space has a warm and tasteful color scheme, with calligraphy artwork and display shelving; the furniture layout supports social interaction and rest, is complemented by indoor plants, and the environment is orderly.	5	D^*^	2	3	2
*S*_18_	A light wood-tone nursing bed, bedside cabinet, desk, and armchair are provided, a television is mounted on the wall, potted plants are placed in the corner, and decorative artwork is hung on the wall.	4		0	0	3
*S*_19_	The interior features large windows with venetian blinds, allowing natural light in.	4	D^*^	−1	−3	−2
*S*_21_	Natural light is brought indoors through doors and windows, ceiling-mounted fixtures provide illumination, and smoke-extraction equipment is installed to ensure ventilation.	4	D^*^	0	−3	0
*S*_6_	The space uses a soft color palette and a moderate proportion of wood finishes, with hanging lanterns and festoon decorations, the surfaces have a matte finish, and the color combinations are moderate in saturation.	3	D^*^	−1	−1	0
*S*_20_	The walls are in light tones, floor-to-ceiling windows ensure ample natural light, and curtains are provided to adjust light levels.	3	D^*^	1	−1	−2
*S*_4_	The space features a fresh and warm color palette, with decorative artwork and bedding, meets residential needs, and is kept orderly.	3	D^*^	0	2	0
*S*_22_	Ceiling-mounted fixtures, recessed downlights, and wall-mounted indirect lighting are provided.	2	D^*^	0	−3	−1
*S*_30_	Display boards and traditional cultural motifs are mounted on the walls, the floor surface is slip-resistant, and lighting is sufficient.	2	D	2	−1	0
*S*_12_	The bathroom walls are finished with small square tiles, towel racks, shelving, and storage containers for daily items are provided, the layout is compact yet functionally complete, and the circulation space can accommodate wheelchair passage.	2	D	0	1	2
*S*_23_	Linear light strips are installed along the wall line of the ceiling together with air diffusers, and the integrated ceiling design improves lighting uniformity.	1	D^*^	0	−4	−1
*S*_17_	The bedroom is furnished with two single beds with the headboards aligned against the wall, and seating and a small round table are placed by the window.	1	D^*^	−3	−4	−1
*S*_24_	The communal activity area is equipped with recessed lighting fixtures and pendant lights, and illumination is sufficient.	1	D^*^	−1	−1	−1
*S*_45_	The pedestrian path runs parallel to the building facade, it features grid-pattern paving with edge bands, and the width allows two-way circulation.	−2	D^*^	3	2	1
*S*_25_	The ground surface is paved with asphalt, which is even and easy to traverse.	−4	D^*^	−2	1	−2
*S*_15_	The site includes paved walkways bordered by shrubs and trees on both sides, vegetation coverage is high and healthy, and high-rise buildings are visible in the distance.	−4	D	1	4	−3
*S*_13_	The outdoor environment features abundant greenery and natural elements, trees and shrubs are well-maintained, and vegetation coverage is high and shows healthy growth.	−5		−4	3	−5
*S*_43_	A vehicular lane with an asphalt surface is provided on one side of the entrance, which forms a boundary with the adjacent pedestrian walkway.	−5		0	−1	−5

^*^Distinguishing statements at P <0.01.

This perspective does not constitute neglect or rejection of the landscape environment, but rather reflects prioritization under resource constraints. Statements concerning building facades, outdoor structures, pathway networks, and high vegetation cover generally rank low within this value orientation (*S*_13_ = −5; *S*_15_ = −4; *S*_25_ = −4; *S*_45_ = −2), indicating that, given limited resources and managerial attention, an institution's external image and landscape are treated as variables with only marginal contribution to day-to-day outcomes in chronic-disease care for older adults. Concurrently, cultural symbols and traditional furnishings are regarded as key elements in fostering a home-like atmosphere within nursing homes. Indoor cultural decorations primarily feature sparse, low-stimulus arrangements that blend into the environmental order (e.g., *S*_41_ = 5; *S*_5_ = 5; *S*_18_ = 4). Familiar interior decorations may evoke memories and strengthen residents' sense of connection and identity (82). Transitioning from home to a collective living environment may trigger loneliness and social disengagement among older adults, exacerbating pre-existing mood disorders (83). Furthermore, interventions that strengthen social support, promote leisure participation, and provide mental health services are crucial for reducing depression prevalence in long-term care facilities (84). This ranking is distinctive relative to the others: for instance, the statement “natural light and uniform illumination” is positively loaded and distinguishing in Factor 1, indicating that this factor emphasizes controllable and stable lighting as foundational conditions. These characteristics collectively form a synergistic perspective of indoor accessibility and low-stimulus environments.

#### 4.3.2 Layout-oriented type: continuous corridors, clear entrances, orderly walking

This perspective centers on a coherent pedestrian network within the site and a clear entrance system. As shown in Supplementary Table S4, the highest-ranked statements focus on functional spaces such as ground-floor external corridors and covered walkways, balancing connectivity with brief stays (*S*_47_ = 5; *S*_52_ = 5). Subsequently, this perspective places significant emphasis on facade order and entrance legibility (*S*_31_ = 4; *S*_33_ = 4), clear spatial boundaries and the organization of barrier-free entry and exit (*S*_46_ = 3; *S*_48_ = 3), as well as pedestrian paths running parallel to building facades with widths adequate for two-way passage (*S*_45_ = 3). Wayfinding details are also emphasized, including wear-resistant and slip-resistant surfaces, color-contrasted wall protection, tactile paving, and pedestrian-vehicle separation (*S*_26_ = 4; *S*_44_ = 2). Cultural displays and signage receive moderately positive ratings (*S*_30_ = 2; *S*_29_ = 2; *S*_28_ = 1), integrated into the identification and wayfinding system rather than treated as standalone decorative elements. Conversely, indoor open-plan activity spaces and centralized rehabilitation equipment receive lower rankings (*S*_49_ = −5; *S*_51_ = −4; *S*_50_ = −3), the value orientation toward “open spatial layouts” is weaker (*S*_39_ = −1; *S*_38_ = −2; *S*_7_ = −2; *S*_37_ = −3), and environments relying solely on mechanical ventilation are rated low (*S*_35_ = −5; *S*_34_ = −4). This evidence indicates that this perspective emphasizes the accessibility, legibility, and maintainability of external corridors, entrance nodes, and the pedestrian system.

From the perspective of chronic disease care, this perspective is internally coherent. Older adults with chronic conditions exhibit reduced endurance and heightened sensitivity to cardiopulmonary strain and weather exposure. Existing research indicates that heat stress can exacerbate pre-existing cardiovascular disease and increase the incidence of acute cardiovascular events among older adults, while windy and snowy conditions significantly curtail outdoor walking by seniors or caregivers (85, 86). Continuous, sheltered walkways or exterior corridors enable cross-building activity and walking under windy, rainy, and sunny conditions, reducing thermal stress and unnecessary detours. Regarding infection control and ventilation, semi-outdoor corridors facilitate adequate air exchange; by increasing ventilation rates, they can effectively reduce the risk of long-range airborne transmission (87), aligning with the emphasis on natural ventilation pathways and the preference for mechanical air supply. Furthermore, this perspective requires low-disturbance, predictable walking surfaces and barrier-free facilities to lower fall risk and maintain gait stability. In addition, prominent landmarks—such as entrance nodes and overhead signage—enhance path legibility, improve older adults' acquisition of spatial memory, and further reduce cross-zone wayfinding costs, consistent with prior scholarship (88). Collectively, this perspective focuses on public settings spanning multiple spaces and time periods, such as corridors, entrance systems, and outdoor road networks. The formation of this category is likely attributable to the breadth of its marginal benefits and clear maintenance protocols, making investments more readily convertible into observable improvements in risk metrics (e.g., slip incidents, disorientation events, cross-area travel time). Consequently, relatively less emphasis is placed on large indoor rehabilitation equipment and open-plan activity spaces, possibly because the benefits of such elements depend on institutional staffing and specialized management, leading to uncertain coverage and usage frequency.

#### 4.3.3 Landscape-centered type: shaded gardens, good greenery, encouraging outdoor stay and social interaction

This orientation indicates that care institutions regard the outdoor built environment and the natural environment as important settings for chronic disease rehabilitation and recovery, as detailed in Supplementary Table S5. The highest-ranked statements emphasize shaded rest areas and continuous, accessible garden corridors (*S*_53_ = 5; *S*_52_ = 5), and further highlight high vegetation cover with healthy tree-shrub systems together with a well-developed pedestrian network (*S*_15_ = 4; *S*_14_ = 4; *S*_354_ = 4). Concurrently, statements prioritizing environmental cleanliness, facilities in good condition, and the integration of buildings and greenery receive consistent support (*S*_1_ = 3; *S*_2_ = 3; *S*_3_ = 1), indicating that this perspective seeks maintainable outdoor basics rather than purely ornamental features. In contrast, indoor elements (ventilation, spatial layout, lighting design, etc.) rank low under this perspective (e.g., *S*_35_ = −5; *S*_28_ = −5; *S*_36_ = −4; *S*_23_ = −4), indirectly indicating that this viewpoint shifts the focus of chronic-disease rehabilitation practices for older adults to the outdoors.

This preference is clearly oriented toward mechanisms for chronic disease rehabilitation. For example, coherent walking routes and distributed resting points help create conditions that support sustained low-intensity activity. Multi-node pavilions and benches break down walking tasks into achievable short-distance goals, thereby encouraging individuals to engage in high-frequency, short-duration, low-intensity outdoor activities, which helps improve outcomes in cardiovascular and metabolic chronic conditions (89). In addition, healthy systems of trees and shrubs and diverse plant communities are consistently rated highly (*S*_13_, *S*_14_, *S*_15_), in line with research on restorative environments showing that natural cues can alleviate tension and negative emotions and enhance attention restoration and emotional stability (90, 91). Older adults who have more contact with green spaces report lower perceived stress levels and experience alleviation of depressive symptoms (92). Exposure to nature may also have a protective effect against schizophrenia, schizotypal personality disorder, and schizoaffective disorder (93). Furthermore, fixed seating, lighting, and permeable enclosures in gardens (*S*_54_ = 4) help create small, recognizable social spaces that can be occupied, thereby encouraging institutional residents to engage in green space activities and family visits, which is crucial for preventing social deterioration and maintaining a sense of autonomy. Consistent with previous research, this study finds that participation in green space activities can enhance the self-worth of older adults with cognitive impairment through meaningful social interaction (94). In summary, this perspective emphasizes outdoor activities and the management of health recovery in older adults with chronic diseases through exposure to nature.

#### 4.3.4 Rehabilitation-driven type: rehabilitation equipment in place, open space, normalized training

This perspective reflects an orientation centered on a functional, barrier-free layout. As shown in Supplementary Table S6, the high-scoring statements emphasize rehabilitation facilities and spacious, accessible spatial configurations (*S*_51_ = 5; *S*_42_ = 5; *S*_8_ = 4; *S*_50_ = 4). On the one hand, this reflects institutions' attention to older adults' chronic-disease rehabilitation and recovery needs, particularly the provision of rehabilitation equipment (e.g., exercise bikes, parallel bars, rehabilitation chairs). As physical therapy has become an integral component of chronic-disease management (95), regular physical-therapy interventions significantly improve pain management, mobility, and overall functional independence in older adults with chronic conditions; this constitutes the standard of care for this population and is crucial for maintaining a satisfactory quality of life (96). On the other hand, unlike Factor 1, which emphasizes accessibility and convenience through lighting, materials, and wayfinding signage, Factor 4 shifts the focus to spatial layout (e.g., *S*_37_ = 4; *S*_50_ = 2). Prior research has emphasized that spatial layout should be clear and legible to facilitate vertical and horizontal navigation, thereby supporting wayfinding for older residents (96). At the same time, accessibility and support across social spaces (e.g., dining, activity, and lounge areas) are improved by more dispersed placement of these spaces (97, 98). Within this orientation, ratings for greenery design and aesthetic functions are relatively low (e.g., *S*_13_ = −5; *S*_32_ = −3), indicating a preference for functionality and practicality. Although greenery and certain aesthetic features benefit older adults' emotional recovery and mental health, Factor 4 prioritizes rehabilitation facilities and barrier-free design over purely visual or environmental aesthetics.

## 5 Conclusion

The contribution of this study lies in proposing a research process for objective Q classification at the institutional level based on signal theory, combining multimodal large language models (MLLM), natural language processing (NLP), and objective Q sorting methods. Automatically generated environmental description text replaces purely manual auditing and subjective rating, which raises the efficiency and consistency of data processing and gives the analysis greater future orientation and practical value. From a perspective that is driven by data and scale, the study identifies environmental orientation types in nursing homes and uses them to build a scalable self testing process and cross institutional benchmarking, while also tracing how the built environment connects to health and rehabilitation. The model complements traditional user centered assessments and helps management align institutional information and communicate regulatory guidance. It has important theoretical and managerial implications.

From a theoretical perspective, this study combines innovative technological methods and the Health-Supportive Environment theory to establish an analytical framework that links environmental representation to orientation types. The results extend Signaling Theory into the field of healthcare environment design, revealing how institutions use self selected environmental images as visual cues in digital media to convey their values regarding the ideal healthcare environment. In doing so, it identifies differences in environmental concepts among nursing institutions and offers a practical basis for design optimization. At the same time, by linking visual representation, semantic structuring, and orientation typification, it turns environmental images into measurable orientation types and offers a reusable framework for subsequent multisource data studies that connect environmental representation with objective scene auditing and relate user perception to health.

From a management and policy perspective, the objective Q sorting workflow developed in this study helps reveal the implicit ranking logic of elderly care institutions in environmental shaping and resource allocation, corrects the mismatch between image presentation and actual needs in chronic disease management, and provides an operational classification framework for family decision making, policy regulation, and environmental design. This in turn improves the efficiency with which limited resources are allocated to environments that support chronic disease management and offers evidence for policy management of core dimensions in medical and elderly care environment design and healthy aging. Specifically, the research generates practice oriented representative statements and several types of management recommendations that highlight priorities for rehabilitation oriented environmental renovation and operational improvement. At the level of concrete choices, Factor 1 can be seen in preferences for less reflective, less saturated surface materials, clearer wayfinding, and handrails that run without gaps. The findings build a methodological bridge between the built environment, medical outcomes, and policy management. These environmental orientation types give families and older adults a clearer way to match their preferences and to compare options across institutions. They also give people in nursing, design, and management a shared language that can work across professional boundaries. The research methodology is transparent and open to audit and has the potential to be applied across multiple fields and regions.

However, the environmental orientations identified in this study through MLLM, NLP, and objective Q methods are mainly oriented toward governance, public communication, and institutional branding. They therefore offer a typological picture of the environmental priorities that institutions choose to project outward and should not be read as a direct assessment of day to day environmental quality or user centered outcomes, nor as a design optimization for specific older subgroups with chronic conditions such as people with Alzheimer's disease or depression. Instead, they sketch a belief map of how nursing institutions rank different built environment elements when they work within limited resources and existing knowledge. The model developed in this study is intended to refine and extend existing environmental audit tools such as TESS-NH, EAT-HC, PEAP, and SCEAM rather than replace them and it helps address the limits of traditional tools that rely on site observation and professional assessors to capture actual environmental characteristics and operating conditions. The wellness and care signals revealed by the findings complement these tools and provide a basis at the institutional level for subsequent field audits.

Compared with other environmental observation scales targeting specific chronic diseases, such as TESS-NH (99) and EAT-HC (100) focused on dementia safety, this study's classification system highlights an institution-oriented model amenable to scalable observation. Compared with the human-centered PEAP (101) and the matrix-based comprehensive assessment tool SCEAM (102) for older adults, this approach focuses on the objective classification and cross-institutional comparison of institutions' built environments, rather than specific environmental assessments of comfort, noise, odor, or real-time processes. In practice, this research process can be used to first identify and define the type and positioning of institutions at the management level, followed by more specific on-site assessments and verification using existing mature auditing tools.

Furthermore, since the environmental images presented by the institutions themselves can only highlight certain spaces, the gap between the signal and reality may exhibit heterogeneity across different institutions and functional zones. Future research could build upon this by combining structured on-site observations, resident and staff experience indicators, and third-party inspection records to conduct triangulation. In addition, future research could consider creating differentiated environments tailored to different functional needs and chronic disease characteristics, integrating individual-level diagnostic and functional assessment data (such as cognitive function, daily living abilities, and emotional state) with risk or wellbeing outcome indicators to more.

## Data Availability

The original contributions presented in the study are included in the article/Supplementary material, further inquiries can be directed to the corresponding authors.
