# Supplementary material for: From images to health insight: integrating MLLM, NLP, and objective Q-sorting of nursing-home built environment orientations
Source: Front Med (Lausanne). 2026 Jan 7;12:1737721. doi: 10.3389/fmed.2025.1737721 (PMC12819831; doi:10.3389/fmed.2025.1737721)
Supplement: Supplementary file 1 [file Supplementary_file_1.docx]

***Supplementary Materials***

**Number of Pages: 24**

**Number of Tables: 6**

**Table of Contents**

[Table S1. Categories of the Q statements. 3](#_Toc21583)

[Table S2. Factor scores for each statement. 7](#_Toc7962)

[Table S3. Rotated factor loadings for the four-factor solution. Bold values indicate Q-sorts that load significantly on each factor. 8](#_Toc2010)

[Table S4. Overview of the defining statements (±4 and 5) and distinguishing statements (at p < 0.05) for factor 2. 17](#_Toc13221)

[Table S5. Overview of the defining statements (±4 and 5) and distinguishing statements (at p < 0.05) for factor 3. 20](#_Toc31185)

[Table S6. Overview of the defining statements (±4 and 5) and distinguishing statements (at p < 0.05) for factor 4. 21](#_Toc6035)

[Text S1: Audit framework for the built environment of nursing homes oriented toward chronic-care and health-supportive rehabilitation for older adults. 23](#_Toc18094)

**Table S1.** Categories of the Q statements.

| **Statement category** | **No.** | **Content of Q Set** |
| --- | --- | --- |
| Outdoor environment and activity accessibility (***C***_1_) | ***S***_1_ | The environment is well maintained and clean, the facilities are intact and orderly, the building is integrated with greenery, and the outdoor space is open, supporting surrounding activities and circulation. |
|  | ***S***_2_ | The environment is properly maintained and clean, the facilities are intact, and the outdoor space can be used for social interaction and rest. |
|  | ***S***_3_ | The exterior facade is tidy, and the fixtures are intact. |
| Interior color scheme and decoration (***C***_2_) | ***S***_4_ | The space features a fresh and warm color palette, with decorative artwork and bedding, meets residential needs, and is kept orderly. |
|  | ***S***_5_ | The space has a warm and tasteful color scheme, with calligraphy artwork and display shelving; the furniture layout supports social interaction and rest, is complemented by indoor plants, and the environment is orderly. |
|  | ***S***_6_ | The space uses a soft color palette and a moderate proportion of wood finishes, with hanging lanterns and festoon decorations, the surfaces have a matte finish, and the color combinations are moderate in saturation. |
| Open-plan living area (***C***_3_) | ***S***_7_ | The layout is open with a wide visual field, it is furnished with mahjong tables, seating, and sofas, the space is spacious and wheelchair-accessible, and it is suitable for group leisure and recreation. |
|  | ***S***_8_ | Display areas are integrated with the corridor space, accessible handrails or wayfinding signage are visible, and the area is open, spacious, and wheelchair-accessible. |
|  | ***S***_9_ | The open living area is spacious and wheelchair-accessible, it presents warm tonal contrasts, it is furnished with a television and indoor plants, the layout supports social interaction and rest, and the environment is orderly. |
| Bathroom configuration (***C***_4_) | ***S***_10_ | The restroom is finished with light-colored tiles, it is equipped with a countertop sink and mirror, a toilet with grab bars on both sides, and a shower area with a shower head, grab bars, and a shower chair. |
|  | ***S***_11_ | The sanitary unit includes a washbasin, a mirrored cabinet, and a toilet, and parts of the wall surface are finished with mosaic tiles. |
|  | ***S***_12_ | The bathroom walls are finished with small square tiles, towel racks, shelving, and storage containers for daily items are provided, the layout is compact yet functionally complete, and the circulation space can accommodate wheelchair passage. |
| Site greenery (***C***_5_) | ***S***_13_ | The outdoor environment features abundant greenery and natural elements, trees and shrubs are well maintained, and vegetation coverage is high and shows healthy growth. |
|  | ***S***_14_ | The site is surrounded by vegetation, including trees and shrubs, which provide shade and visual screening. |
|  | ***S***_15_ | The site includes paved walkways bordered by shrubs and trees on both sides, vegetation coverage is high and healthy, and high-rise buildings are visible in the distance. |
| Care bed arrangement (***C***_6_) | ***S***_16_ | The dedicated functional area is furnished with a nursing bed and a bedside cabinet, bedding is complete, and daily-use items such as a fan are placed beside the bed. |
|  | ***S***_17_ | The bedroom is furnished with two single beds with the headboards aligned against the wall, and seating and a small round table are placed by the window. |
|  | ***S***_18_ | A light wood-tone nursing bed, bedside cabinet, desk, and armchair are provided, a television is mounted on the wall, potted plants are placed in the corner, and decorative artwork is hung on the wall. |
| Natural lighting and window arrangement (***C***_7_) | ***S***_19_ | The interior features large windows with venetian blinds, allowing natural light in. |
|  | ***S***_20_ | The walls are in light tones, floor-to-ceiling windows ensure ample natural light, and curtains are provided to adjust light levels. |
|  | ***S***_21_ | Natural light is brought indoors through doors and windows, ceiling-mounted fixtures provide illumination, and smoke-extraction equipment is installed to ensure ventilation. |
| Lighting system (***C***_8_) | ***S***_22_ | Ceiling-mounted fixtures, recessed downlights, and wall-mounted indirect lighting are provided. |
|  | ***S***_23_ | Linear light strips are installed along the wall line of the ceiling together with air diffusers, and the integrated ceiling design improves lighting uniformity. |
|  | ***S***_24_ | The communal activity area is equipped with recessed lighting fixtures and pendant lights, and illumination is sufficient. |
| Surface paving (***C***_9_) | ***S***_25_ | The ground surface is paved with asphalt, which is even and easy to traverse. |
|  | ***S***_26_ | The floor surface is finished with a wear-resistant coating, and the wall surface features a two-tone protective wall treatment. |
|  | ***S***_27_ | The ground surface is paved with regular square tiles, and the evenness is good. |
| Surface decoration and wayfinding signage (***C***_10_) | ***S***_28_ | Decorative artwork and informational signage are affixed to the walls. |
|  | ***S***_29_ | Decorative hanging ornaments are suspended from the ceiling, cultural slogans and wall decorations are displayed on the walls, the color scheme is vivid, and indoor plants are provided. |
|  | ***S***_30_ | Display boards and traditional cultural motifs are mounted on the walls, the floor surface is slip-resistant, and lighting is sufficient. |
| Building facade (***C***_11_) | ***S***_31_ | The main exterior facade of the building is in light tones, the decorative style is simple, and the structure is multi-story. |
|  | ***S***_32_ | The main building facade is primarily light-colored and in a neo-Chinese decorative style, the roof is tiled, and the windows are aligned in an orderly manner. |
|  | ***S***_33_ | The main facade uses faux-brick finishes and brick-pattern cladding, the decorative style is simple, and columns support an eave corridor. |
| Ventilation and air-conditioning system (***C***_12_) | ***S***_34_ | Ventilation is achieved through operable external windows together with air conditioning. |
|  | ***S***_35_ | Air conditioning is supplied through ceiling-mounted air diffusers. |
|  | ***S***_36_ | Ventilation is provided by ceiling-mounted exhaust together with operable window sashes. |
| Spatial scale and wheelchair circulation (***C***_13_) | ***S***_37_ | The space is wide, and wheelchair circulation is possible. |
|  | ***S***_38_ | A relatively independent interior functional area has a wide visual field, the space is generous and wheelchair-accessible. |
|  | ***S***_39_ | The open spatial layout includes a relatively long corridor with a wide line of sight, the space is broad and supports smooth wheelchair passage. |
| Public-area seating and reception desk arrangement (***C***_14_) | ***S***_40_ | Sets of tables and chairs are arranged in the common area, forming a social seating layout. |
|  | ***S***_41_ | Sofas and a wood reception counter are provided, shelving is placed nearby, illuminated lettering is installed on the wall, accessible handrails are installed along the corridor, the floor has a matte finish, the proportion of wood finishes is moderate, and the color scheme is low in saturation. |
|  | ***S***_42_ | Round dining tables with rotating trays and chairs are provided, together with a plant wall and a service counter, and the area is spacious and wheelchair-accessible. |
| Entry roadway and pedestrian interface (***C***_15_) | ***S***_43_ | A vehicular lane with an asphalt surface is provided on one side of the entrance, which forms a boundary with the adjacent pedestrian walkway. |
|  | ***S***_44_ | At the outdoor entrance, the pedestrian walkway and the vehicular lane are separated by a curb, and tactile paving for accessibility is installed along the curb. |
|  | ***S***_45_ | The pedestrian path runs parallel to the building facade, it features grid-pattern paving with edge bands, and the width allows two-way circulation. |
| Entrance structure (***C***_16_) | ***S***_46_ | At the corner, an arched entrance and a gated wall opening are provided, and the entrance includes fencing and a header sign. |
|  | ***S***_47_ | The ground-floor exterior corridor includes openings and equipment access doors, parts of the facade are finished with stone cladding, and the line of sight terminates at an activity node within the courtyard. |
|  | ***S***_48_ | The entrance is equipped with double doors and an accessible ramp in front, and handrails are installed on both sides of the ramp. |
| Rehabilitation equipment (***C***_17_) | ***S***_49_ | The layout is open with a wide visual field, multiple sets of rehabilitation and exercise equipment and suspended training devices are provided. |
|  | ***S***_50_ | The indoor rehabilitation area is equipped with treadmills, parallel bars, steps and ramps, and stationary cycling equipment, and activity subzones are designated. |
|  | ***S***_51_ | The interior is furnished with multiple rehabilitation devices (training carts, parallel bars, rehabilitation chairs) and with tables and chairs. |
| Shaded outdoor circulation and resting nodes (***C***_18_) | ***S***_52_ | An outdoor shaded corridor with a transparent roof, using a metal frame and translucent materials, is provided, with a tiled pedestrian walkway beneath it, flowerbeds and a variety of greenery including trees and shrubs alongside, the environment is orderly, and the corridor provides outdoor circulation and a place to rest. |
|  | ***S***_53_ | A wooden pavilion with a tiled roof is installed under tree shade, with a stone-paved ground surface below, and surrounding trees, fencing, and steps, the environment is orderly, and the pavilion provides a shaded outdoor place to stop and rest. |
|  | ***S***_54_ | In the outdoor garden, fixed outdoor seating and lighting poles are visible, the path is finished with wood decking, and a transparent guardrail is installed alongside. |

**Table S2**. Factor scores for each statement.

| **No.** | **F1 Z-score** | **F1 Rank** | **F2 Z-score** | **F2 Rank** | **F3 Z-score** | **F3 Rank** | **F4 Z-score** | **F4 Rank** |
| --- | --- | --- | --- | --- | --- | --- | --- | --- |
| ***S***_1_ | -0.59 | 40 | 0.13 | 24 | 1.33 | 7 | -0.93 | 45 |
| ***S***_2_ | -0.02 | 27 | -0.26 | 37 | 1.34 | 6 | 0.24 | 22 |
| ***S***_3_ | -0.31 | 36 | 0.22 | 19 | 0.45 | 20 | -0.59 | 39 |
| ***S***_4_ | 1.02 | 10 | 0.09 | 26 | 0.75 | 12 | -0.17 | 29 |
| ***S***_5_ | 1.72 | 2 | 0.7 | 16 | 1.21 | 9 | 0.71 | 16 |
| ***S***_6_ | 1.41 | 6 | -0.14 | 33 | -0.26 | 33 | 0.1 | 25 |
| ***S***_7_ | 0.69 | 12 | -0.91 | 43 | 0.6 | 16 | 1.21 | 6 |
| ***S***_8_ | 0.39 | 21 | 0.15 | 23 | 0.19 | 24 | 1.63 | 3 |
| ***S***_9_ | 0.61 | 16 | -0.28 | 38 | 1.23 | 8 | 0.94 | 14 |
| ***S***_10_ | 0.08 | 26 | -0.74 | 40 | -0.86 | 43 | 0.32 | 20 |
| ***S***_11_ | -0.29 | 35 | -1.1 | 44 | -1.29 | 47 | -0.04 | 27 |
| ***S***_12_ | 0.62 | 15 | -0.05 | 31 | 0.29 | 23 | 1.07 | 13 |
| ***S***_13_ | -1.88 | 53 | -1.57 | 51 | 1.15 | 10 | -2.05 | 54 |
| ***S***_14_ | -0.25 | 33 | 0.19 | 21 | 1.65 | 4 | -0.45 | 37 |
| ***S***_15_ | -1.49 | 51 | 0.32 | 18 | 1.7 | 3 | -1.04 | 46 |
| ***S***_16_ | -0.03 | 28 | -1.41 | 49 | -0.7 | 42 | 0.29 | 21 |
| ***S***_17_ | 0.49 | 19 | -1.17 | 45 | -1.63 | 51 | -0.3 | 34 |
| ***S***_18_ | 1.53 | 3 | -0.01 | 30 | 0.08 | 26 | 1.17 | 9 |
| ***S***_19_ | 1.46 | 4 | -0.07 | 32 | -1.32 | 49 | -0.59 | 40 |
| ***S***_20_ | 1.15 | 9 | 0.67 | 17 | -0.61 | 38 | -0.78 | 42 |
| ***S***_21_ | 1.42 | 5 | 0.1 | 25 | -0.98 | 45 | -0.23 | 30 |
| ***S***_22_ | 0.78 | 11 | 0.02 | 27 | -1.3 | 48 | -0.3 | 33 |
| ***S***_23_ | 0.5 | 18 | 0.01 | 29 | -1.66 | 52 | -0.38 | 35 |
| ***S***_24_ | 0.35 | 22 | -0.21 | 34 | -0.45 | 35 | -0.44 | 36 |
| ***S***_25_ | -1.44 | 50 | -0.31 | 39 | 0.45 | 19 | -0.78 | 43 |
| ***S***_26_ | -0.19 | 32 | 1.29 | 5 | 0.79 | 11 | -0.02 | 26 |
| ***S***_27_ | -1.29 | 47 | 0.19 | 22 | 0.17 | 25 | -1.11 | 47 |
| ***S***_28_ | -0.34 | 39 | 0.19 | 20 | -1.77 | 54 | -0.49 | 38 |
| ***S***_29_ | -0.05 | 30 | 0.9 | 12 | 0.38 | 21 | -0.76 | 41 |
| ***S***_30_ | 0.64 | 14 | 0.99 | 11 | -0.24 | 32 | 0.12 | 24 |
| ***S***_31_ | 0.16 | 25 | 1.58 | 3 | 0.67 | 13 | -0.25 | 31 |
| ***S***_32_ | -0.33 | 37 | 0.79 | 14 | -0.69 | 41 | -1.27 | 48 |
| ***S***_33_ | -0.34 | 38 | 1.31 | 4 | -0.36 | 34 | -0.25 | 32 |
| ***S***_34_ | -1.17 | 45 | -1.51 | 50 | -1.25 | 46 | -1.55 | 51 |
| ***S***_35_ | -1.85 | 52 | -1.97 | 54 | -1.68 | 53 | -1.82 | 52 |
| ***S***_36_ | -1.27 | 46 | -1.2 | 46 | -1.53 | 50 | -1.47 | 50 |
| ***S***_37_ | -0.28 | 34 | -1.27 | 48 | -0.09 | 28 | 1.09 | 11 |
| ***S***_38_ | 0.59 | 17 | -0.89 | 42 | -0.11 | 29 | 1.19 | 8 |
| ***S***_39_ | -0.03 | 29 | -0.25 | 36 | 0.32 | 22 | 1.13 | 10 |
| ***S***_40_ | 0.43 | 20 | -0.83 | 41 | -0.11 | 30 | 0.8 | 15 |
| ***S***_41_ | 1.94 | 1 | 1.04 | 10 | 0.65 | 14 | 1.22 | 5 |
| ***S***_42_ | 1.32 | 7 | -0.24 | 35 | 0.53 | 17 | 1.72 | 2 |
| ***S***_43_ | -2.06 | 54 | 0.01 | 28 | -0.52 | 36 | -1.85 | 53 |
| ***S***_44_ | -1.4 | 49 | 0.84 | 13 | -0.2 | 31 | -1.33 | 49 |
| ***S***_45_ | -0.7 | 41 | 1.05 | 9 | 0.63 | 15 | 0.18 | 23 |
| ***S***_46_ | -1.06 | 44 | 1.25 | 6 | -0.9 | 44 | -0.87 | 44 |
| ***S***_47_ | 0.29 | 23 | 2.01 | 1 | 0.49 | 18 | 0.43 | 18 |
| ***S***_48_ | -0.74 | 42 | 1.24 | 7 | -0.67 | 40 | -0.17 | 28 |
| ***S***_49_ | -1.36 | 48 | -1.83 | 53 | -0.53 | 37 | 1.08 | 12 |
| ***S***_50_ | -0.92 | 43 | -1.23 | 47 | 0.06 | 27 | 1.59 | 4 |
| ***S***_51_ | -0.06 | 31 | -1.79 | 52 | -0.62 | 39 | 1.85 | 1 |
| ***S***_52_ | 1.25 | 8 | 1.95 | 2 | 1.84 | 2 | 1.2 | 7 |
| ***S***_53_ | 0.2 | 24 | 0.79 | 15 | 1.86 | 1 | 0.42 | 19 |
| ***S***_54_ | 0.68 | 13 | 1.23 | 8 | 1.55 | 5 | 0.6 | 17 |

**Table S3**. Rotated factor loadings for the four-factor solution. Bold values indicate Q-sorts that load significantly on each factor.

| **Nm** | **Q sort** | **Factor Loadings** | | | | |
| --- | --- | --- | --- | --- | --- | --- |
|  |  | **Factor 1** | **Factor 2** | **Factor 3** | **Factor 4** | **Assigned Factor** |
| 1 | P001 | **0.561** | 0.016 | 0.293 | 0.296 | F1 |
| 2 | P002 | -0.159 | **0.695** | 0.616 | 0.080 | F2 |
| 3 | P003 | 0.105 | 0.609 | 0.673 | 0.281 | - |
| 4 | P004 | 0.342 | -0.321 | 0.409 | **0.623** | F4 |
| 5 | P005 | -0.093 | **0.842** | 0.310 | 0.178 | F2 |
| 6 | P006 | -0.203 | **0.747** | 0.238 | -0.134 | F2 |
| 7 | P007 | 0.148 | **0.852** | 0.247 | -0.155 | F2 |
| 8 | P008 | 0.334 | 0.457 | **0.659** | 0.274 | F3 |
| 9 | P009 | 0.079 | **0.851** | 0.115 | -0.231 | F2 |
| 10 | P010 | 0.434 | **0.712** | -0.107 | 0.297 | F2 |
| 11 | P011 | **0.909** | -0.043 | 0.071 | -0.056 | F1 |
| 12 | P012 | **0.641** | 0.516 | -0.021 | 0.187 | F1 |
| 13 | P013 | 0.310 | **0.856** | -0.096 | 0.210 | F2 |
| 14 | P014 | 0.251 | -0.326 | **0.677** | 0.486 | F3 |
| 15 | P015 | 0.348 | -0.158 | **0.573** | 0.306 | F3 |
| 16 | P016 | **0.763** | 0.522 | 0.044 | 0.047 | F1 |
| 17 | P017 | 0.203 | **0.874** | 0.011 | -0.101 | F2 |
| 18 | P018 | **0.918** | 0.148 | -0.139 | -0.126 | F1 |
| 19 | P019 | 0.135 | **0.886** | -0.187 | -0.233 | F2 |
| 20 | P020 | -0.276 | **0.774** | 0.277 | 0.195 | F2 |
| 21 | P021 | **0.875** | -0.068 | 0.223 | 0.335 | F1 |
| 22 | P022 | **0.825** | -0.064 | 0.407 | 0.130 | F1 |
| 23 | P023 | 0.495 | 0.118 | 0.564 | 0.459 | - |
| 24 | P024 | 0.665 | 0.629 | -0.249 | 0.076 | - |
| 25 | P025 | 0.883 | -0.011 | 0.228 | 0.330 | F1 |
| 26 | P026 | 0.307 | **0.673** | 0.307 | -0.268 | F2 |
| 27 | P027 | 0.929 | 0.076 | 0.028 | -0.144 | F1 |
| 28 | P028 | 0.818 | -0.267 | 0.300 | 0.156 | F1 |
| 29 | P029 | 0.956 | 0.100 | 0.025 | -0.004 | F1 |
| 30 | P030 | 0.905 | -0.065 | 0.248 | 0.160 | F1 |
| 31 | P031 | 0.454 | **0.792** | -0.068 | -0.153 | F2 |
| 32 | P032 | 0.905 | 0.000 | -0.147 | -0.026 | F1 |
| 33 | P033 | 0.714 | -0.337 | 0.299 | 0.410 | F1 |
| 34 | P034 | 0.120 | **0.690** | 0.658 | -0.006 | F2 |
| 35 | P035 | 0.124 | **0.863** | 0.337 | -0.280 | F2 |
| 36 | P036 | 0.223 | 0.491 | **0.664** | 0.165 | F3 |
| 37 | P037 | **0.766** | 0.074 | 0.414 | 0.366 | F1 |
| 38 | P038 | 0.059 | 0.310 | **0.845** | 0.112 | F3 |
| 39 | P039 | 0.271 | **0.734** | 0.529 | -0.183 | F2 |
| 40 | P040 | **0.912** | -0.053 | 0.209 | 0.086 | F1 |
| 41 | P041 | **0.924** | -0.143 | 0.243 | 0.025 | F1 |
| 42 | P042 | **0.888** | 0.250 | 0.086 | 0.222 | F1 |
| 43 | P043 | **0.754** | 0.368 | 0.360 | -0.280 | F1 |
| 44 | P044 | 0.202 | **0.670** | 0.558 | 0.018 | F2 |
| 45 | P045 | -0.195 | 0.627 | 0.649 | -0.027 | - |
| 46 | P046 | **0.771** | 0.544 | -0.065 | -0.032 | F1 |
| 47 | P047 | **0.862** | -0.035 | 0.257 | 0.224 | F1 |
| 48 | P048 | **0.956** | 0.113 | 0.043 | -0.001 | F1 |
| 49 | P049 | **0.911** | -0.070 | 0.196 | 0.119 | F1 |
| 50 | P050 | **0.932** | 0.131 | 0.148 | -0.022 | F1 |
| 51 | P051 | **0.938** | -0.086 | 0.023 | -0.127 | F1 |
| 52 | P052 | **0.948** | 0.214 | 0.011 | -0.055 | F1 |
| 53 | P053 | **0.947** | 0.204 | -0.013 | -0.013 | F1 |
| 54 | P054 | **0.891** | 0.230 | -0.290 | -0.069 | F1 |
| 55 | P055 | 0.320 | 0.301 | **0.806** | -0.203 | F3 |
| 56 | P056 | **0.885** | 0.316 | -0.091 | -0.005 | F1 |
| 57 | P057 | **0.849** | 0.029 | -0.175 | -0.091 | F1 |
| 58 | P058 | -0.170 | **0.742** | 0.438 | 0.024 | F2 |
| 59 | P059 | **0.816** | 0.439 | -0.014 | -0.081 | F1 |
| 60 | P060 | **0.848** | 0.275 | -0.268 | -0.174 | F1 |
| 61 | P061 | **0.767** | 0.447 | -0.039 | -0.007 | F1 |
| 62 | P062 | -0.032 | 0.333 | **0.880** | 0.051 | F3 |
| 63 | P063 | 0.020 | 0.494 | **0.760** | -0.030 | F3 |
| 64 | P064 | -0.202 | **0.716** | 0.355 | 0.254 | F2 |
| 65 | P065 | -0.084 | **0.830** | 0.285 | 0.247 | F2 |
| 66 | P066 | 0.546 | -0.218 | 0.103 | 0.535 | - |
| 67 | P067 | 0.328 | 0.565 | 0.578 | 0.168 | - |
| 68 | P068 | -0.147 | 0.163 | **0.931** | 0.073 | F3 |
| 69 | P069 | 0.050 | **0.680** | 0.560 | 0.153 | F2 |
| 70 | P070 | **0.864** | 0.066 | 0.270 | 0.327 | F1 |
| 71 | P071 | **0.937** | 0.064 | 0.160 | -0.010 | F1 |
| 72 | P072 | **0.890** | 0.312 | 0.057 | 0.004 | F1 |
| 73 | P073 | **0.953** | 0.079 | -0.100 | -0.038 | F1 |
| 74 | P074 | **0.932** | -0.043 | 0.221 | 0.087 | F1 |
| 75 | P075 | **0.946** | 0.129 | -0.025 | 0.064 | F1 |
| 76 | P076 | 0.041 | -0.034 | **0.941** | 0.145 | - |
| 77 | P077 | 0.117 | 0.001 | **0.935** | 0.157 | - |
| 78 | P078 | 0.686 | 0.239 | 0.307 | 0.443 | F1 |
| 79 | P079 | 0.232 | **0.864** | 0.324 | 0.043 | F2 |
| 80 | P080 | 0.205 | **0.800** | 0.361 | -0.280 | F2 |
| 81 | P081 | 0.393 | **0.675** | 0.330 | -0.389 | F2 |
| 82 | P082 | 0.510 | **0.734** | 0.138 | -0.275 | F2 |
| 83 | P083 | **0.932** | 0.070 | 0.066 | 0.058 | F1 |
| 84 | P084 | **0.751** | -0.219 | 0.476 | 0.283 | F1 |
| 85 | P085 | 0.545 | -0.014 | 0.336 | 0.541 | - |
| 86 | P086 | **0.740** | 0.574 | -0.096 | 0.026 | F1 |
| 87 | P087 | 0.288 | **0.838** | 0.277 | -0.259 | F2 |
| 88 | P088 | -0.041 | 0.475 | **0.791** | -0.021 | F3 |
| 89 | P089 | **0.828** | 0.156 | 0.342 | 0.269 | F1 |
| 90 | P090 | -0.022 | 0.064 | **0.724** | 0.392 | F3 |
| 91 | P091 | **0.712** | 0.509 | 0.058 | 0.250 | F1 |
| 92 | P092 | **0.927** | 0.193 | 0.040 | -0.130 | F1 |
| 93 | P093 | 0.266 | **0.824** | 0.323 | -0.242 | F2 |
| 94 | P094 | **0.784** | 0.513 | -0.135 | 0.108 | F1 |
| 95 | P095 | **0.900** | 0.063 | 0.235 | 0.152 | F1 |
| 96 | P096 | 0.448 | 0.278 | -0.338 | 0.288 | - |
| 97 | P097 | **0.651** | -0.300 | -0.074 | 0.488 | F1 |
| 98 | P098 | 0.279 | **0.875** | 0.237 | -0.172 | F2 |
| 99 | P099 | **0.963** | 0.060 | 0.015 | -0.068 | F1 |
| 100 | P100 | **0.838** | 0.157 | -0.100 | -0.226 | F1 |
| 101 | P101 | **0.760** | -0.055 | 0.436 | 0.381 | F1 |
| 102 | P102 | **0.874** | 0.325 | -0.072 | -0.045 | F1 |
| 103 | P103 | 0.513 | **0.747** | -0.251 | 0.015 | F2 |
| 104 | P104 | **0.732** | 0.582 | -0.089 | -0.206 | F1 |
| 105 | P105 | 0.565 | **0.738** | -0.138 | 0.017 | F2 |
| 106 | P106 | 0.057 | 0.297 | **0.686** | 0.368 | F3 |
| 107 | P107 | -0.036 | **0.816** | 0.492 | -0.107 | F2 |
| 108 | P108 | **0.935** | -0.008 | 0.179 | 0.175 | F1 |
| 109 | P109 | **0.768** | 0.458 | -0.205 | -0.077 | F1 |
| 110 | P110 | 0.488 | -0.126 | **0.740** | 0.336 | F3 |
| 111 | P111 | **0.820** | -0.020 | 0.014 | 0.130 | F1 |
| 112 | P112 | 0.289 | -0.067 | **0.844** | 0.171 | F3 |
| 113 | P113 | -0.037 | 0.504 | **0.698** | 0.151 | F3 |
| 114 | P114 | -0.242 | 0.578 | **0.679** | 0.145 | F3 |
| 115 | P115 | 0.549 | 0.649 | -0.401 | -0.108 | - |
| 116 | P116 | **0.962** | 0.079 | 0.031 | -0.023 | F1 |
| 117 | P117 | 0.425 | 0.594 | 0.316 | -0.488 | - |
| 118 | P118 | 0.213 | **0.893** | -0.193 | -0.110 | F2 |
| 119 | P119 | **0.913** | 0.200 | -0.133 | 0.032 | F1 |
| 120 | P120 | 0.324 | **0.839** | 0.298 | -0.197 | F2 |
| 121 | P121 | -0.009 | **0.958** | -0.127 | 0.016 | F2 |
| 122 | P122 | 0.541 | 0.614 | 0.194 | -0.328 | - |
| 123 | P123 | -0.039 | 0.633 | **0.695** | 0.035 | F3 |
| 124 | P124 | **0.662** | 0.555 | 0.010 | 0.207 | F1 |
| 125 | P125 | **0.888** | 0.364 | -0.083 | 0.036 | F1 |
| 126 | P126 | -0.074 | **0.903** | 0.201 | 0.075 | F2 |
| 127 | P127 | 0.049 | 0.543 | **0.754** | -0.041 | F3 |
| 128 | P128 | **0.894** | 0.021 | 0.276 | 0.153 | F1 |
| 129 | P129 | -0.090 | **0.792** | -0.023 | 0.350 | F2 |
| 130 | P130 | **0.925** | 0.037 | 0.233 | 0.178 | F1 |
| 131 | P131 | 0.037 | 0.516 | 0.658 | -0.434 | - |
| 132 | P132 | **0.886** | 0.282 | -0.073 | 0.048 | F1 |
| 133 | P133 | 0.047 | **0.807** | 0.404 | -0.089 | F2 |
| 134 | P134 | 0.452 | **0.795** | 0.192 | -0.016 | F2 |
| 135 | P135 | **0.923** | 0.013 | 0.244 | 0.069 | F1 |
| 136 | P136 | -0.157 | **0.713** | 0.573 | -0.019 | F2 |
| 137 | P137 | 0.047 | **0.807** | 0.404 | -0.089 | F2 |
| 138 | P138 | **0.904** | 0.265 | -0.133 | -0.108 | F1 |
| 139 | P139 | -0.286 | **0.894** | 0.163 | 0.012 | F2 |
| 140 | P140 | **0.955** | 0.098 | 0.100 | 0.084 | F1 |
| 141 | P141 | 0.069 | 0.520 | 0.658 | -0.451 | - |
| 142 | P142 | **0.772** | 0.336 | -0.169 | -0.137 | F1 |
| 143 | P143 | 0.067 | 0.540 | **0.731** | -0.016 | F3 |
| 144 | P144 | 0.120 | 0.339 | **0.870** | -0.187 | F3 |
| 145 | P145 | 0.150 | 0.606 | 0.527 | -0.505 | - |
| 146 | P146 | 0.154 | **0.724** | 0.500 | -0.385 | F2 |
| 147 | P147 | **0.930** | 0.243 | -0.096 | 0.041 | F1 |
| 148 | P148 | **0.791** | 0.094 | 0.477 | 0.281 | F1 |
| 149 | P149 | **0.621** | 0.483 | -0.270 | 0.095 | F1 |
| 150 | P150 | 0.196 | 0.596 | **0.685** | -0.273 | F3 |
| 151 | P151 | **0.716** | 0.368 | -0.100 | 0.133 | F1 |
| 152 | P152 | **0.938** | 0.095 | -0.083 | -0.111 | F1 |
| 153 | P153 | 0.640 | 0.581 | -0.318 | -0.044 | - |
| 154 | P154 | **0.932** | 0.130 | 0.054 | -0.053 | F1 |
| 155 | P155 | 0.411 | **0.699** | 0.305 | -0.402 | F2 |
| 156 | P156 | **0.828** | 0.188 | 0.284 | 0.145 | F1 |
| 157 | P157 | **0.886** | 0.148 | -0.057 | -0.166 | F1 |
| 158 | P158 | 0.209 | **0.900** | -0.132 | -0.019 | F2 |
| 159 | P159 | 0.016 | 0.107 | **0.862** | -0.319 | F3 |
| 160 | P160 | 0.039 | **0.877** | 0.295 | -0.080 | F2 |
| 161 | P161 | **0.920** | 0.162 | 0.109 | -0.116 | F1 |
| 162 | P162 | **0.949** | 0.068 | 0.075 | -0.046 | F1 |
| 163 | P163 | 0.557 | -0.310 | 0.374 | 0.519 | - |
| 164 | P164 | 0.287 | -0.078 | **0.665** | 0.515 | F3 |
| 165 | P165 | -0.110 | 0.538 | **0.765** | -0.155 | F3 |
| 166 | P166 | 0.136 | **0.860** | 0.283 | -0.247 | F2 |
| 167 | P167 | **0.899** | 0.048 | 0.304 | -0.055 | F1 |
| 168 | P168 | 0.458 | **0.799** | 0.142 | -0.167 | F2 |
| 169 | P169 | **0.901** | 0.221 | 0.007 | -0.184 | F1 |
| 170 | P170 | **0.905** | -0.043 | 0.210 | 0.122 | F1 |
| 171 | P171 | 0.045 | **0.722** | 0.502 | -0.278 | F2 |
| 172 | P172 | 0.631 | **0.687** | -0.033 | -0.197 | F2 |
| 173 | P173 | 0.124 | **0.844** | 0.380 | -0.212 | F2 |
| 174 | P174 | 0.331 | 0.465 | 0.454 | 0.346 | - |
| 175 | P175 | **0.682** | 0.518 | 0.051 | -0.302 | F1 |
| 176 | P176 | **0.836** | 0.257 | -0.167 | -0.027 | F1 |
| 177 | P177 | **0.766** | 0.439 | 0.012 | 0.247 | F1 |
| 178 | P178 | **0.898** | -0.057 | 0.136 | 0.214 | F1 |
| 179 | P179 | -0.149 | 0.247 | **0.885** | 0.107 | F3 |
| 180 | P180 | **0.880** | 0.049 | 0.164 | 0.266 | F1 |
| 181 | P181 | **0.884** | 0.043 | 0.053 | 0.280 | F1 |
| 182 | P182 | 0.346 | 0.439 | **0.782** | -0.201 | F3 |
| 183 | P183 | 0.215 | **0.913** | -0.166 | -0.052 | F2 |
| 184 | P184 | **0.710** | -0.182 | 0.074 | 0.334 | F1 |
| 185 | P185 | **0.877** | 0.219 | -0.171 | -0.312 | F1 |
| 186 | P186 | 0.222 | **0.938** | -0.147 | 0.071 | F2 |
| 187 | P187 | **0.935** | 0.201 | 0.030 | 0.012 | F1 |
| 188 | P188 | 0.635 | 0.620 | 0.121 | -0.062 | - |
| 189 | P189 | **0.772** | 0.437 | -0.043 | 0.217 | F1 |
| 190 | P190 | **0.942** | 0.024 | 0.184 | 0.091 | F1 |
| 191 | P191 | **0.900** | 0.100 | 0.237 | -0.138 | F1 |
| 192 | P192 | 0.025 | 0.104 | **0.949** | -0.069 | F3 |
| 193 | P193 | **0.955** | 0.032 | 0.021 | 0.039 | F1 |
| 194 | P194 | 0.149 | **0.884** | 0.231 | -0.068 | F2 |
| 195 | P195 | **0.672** | 0.040 | 0.593 | 0.069 | F1 |
| 196 | P196 | **0.837** | -0.009 | 0.281 | 0.342 | F1 |
| 197 | P197 | **0.946** | 0.069 | 0.016 | -0.054 | F1 |
| 198 | P198 | **0.899** | -0.171 | 0.156 | 0.294 | F1 |
| 199 | P199 | **0.899** | -0.171 | 0.156 | 0.294 | F1 |
| 200 | P200 | **0.929** | 0.210 | 0.007 | 0.102 | F1 |
| 201 | P201 | **0.920** | 0.213 | 0.068 | 0.060 | F1 |
| 202 | P202 | **0.753** | 0.147 | 0.153 | 0.156 | F1 |
| 203 | P203 | **0.740** | 0.065 | 0.538 | 0.132 | F1 |
| 204 | P204 | **0.718** | 0.422 | 0.127 | 0.350 | F1 |
| 205 | P205 | -0.039 | 0.384 | **0.866** | -0.214 | F3 |
| 206 | P206 | **0.821** | -0.008 | 0.435 | 0.088 | F1 |
| 207 | P207 | 0.485 | **0.693** | 0.289 | -0.284 | F2 |
| 208 | P208 | **0.946** | -0.023 | -0.005 | 0.187 | F1 |
| 209 | P209 | 0.620 | 0.638 | -0.125 | 0.207 | - |
| 210 | P210 | **0.936** | 0.079 | 0.021 | 0.184 | F1 |
| 211 | P211 | -0.039 | **0.951** | 0.045 | 0.107 | F2 |
| 212 | P212 | -0.062 | 0.114 | **0.929** | 0.045 | F3 |
| 213 | P213 | 0.270 | **0.802** | 0.283 | -0.222 | F2 |
| 214 | P214 | 0.221 | **0.841** | 0.250 | -0.148 | F2 |
| 215 | P215 | **0.809** | -0.180 | 0.408 | 0.309 | F1 |
| 216 | P216 | 0.459 | **0.830** | -0.010 | 0.032 | F2 |
| 217 | P217 | **0.938** | 0.095 | 0.032 | 0.159 | F1 |
| 218 | P218 | **0.926** | -0.098 | 0.256 | 0.098 | F1 |
| 219 | P219 | **0.884** | 0.249 | 0.001 | -0.009 | F1 |
| 220 | P220 | 0.326 | **0.878** | -0.138 | -0.112 | F2 |
| 221 | P221 | **0.920** | 0.307 | -0.039 | 0.000 | F1 |
| 222 | P222 | 0.235 | **0.854** | 0.277 | -0.047 | F2 |
| 223 | P223 | **0.820** | 0.123 | -0.072 | 0.032 | F1 |
| 224 | P224 | 0.317 | **0.917** | -0.082 | -0.013 | F2 |
| 225 | P225 | 0.033 | **0.784** | 0.522 | 0.047 | F2 |
| 226 | P226 | **0.855** | 0.129 | -0.140 | -0.231 | F1 |
| 227 | P227 | 0.632 | **0.678** | 0.086 | -0.219 | F2 |
| 228 | P228 | 0.283 | 0.593 | 0.603 | -0.332 | - |
| 229 | P229 | **0.854** | 0.282 | 0.149 | 0.084 | F1 |
| 230 | P230 | 0.309 | **0.758** | 0.418 | 0.013 | F2 |
| 231 | P231 | -0.027 | 0.200 | **0.931** | -0.025 | F3 |
| 232 | P232 | **0.921** | 0.110 | 0.139 | 0.003 | F1 |
| 233 | P233 | 0.270 | 0.658 | 0.508 | -0.390 | - |
| 234 | P234 | 0.226 | **0.887** | 0.153 | -0.229 | F2 |
| 235 | P235 | 0.396 | 0.594 | 0.451 | 0.319 | - |
| 236 | P236 | **0.900** | -0.008 | 0.156 | 0.103 | F1 |
| 237 | P237 | **0.921** | 0.287 | -0.063 | -0.112 | F1 |
| 238 | P238 | 0.221 | **0.805** | 0.268 | -0.412 | F2 |
| 239 | P239 | **0.865** | 0.311 | 0.061 | -0.049 | F1 |
| 240 | P240 | 0.163 | **0.872** | 0.193 | 0.166 | F2 |
| 241 | P241 | **0.755** | 0.319 | 0.261 | 0.300 | F1 |
| 242 | P242 | **0.812** | 0.038 | 0.117 | -0.010 | F1 |
| 243 | P243 | **0.680** | 0.178 | 0.053 | 0.324 | F1 |
| 244 | P244 | -0.018 | **0.812** | 0.354 | -0.378 | F2 |
| 245 | P245 | 0.926 | 0.127 | -0.011 | -0.147 | F1 |
| 246 | P246 | 0.888 | 0.309 | 0.034 | -0.145 | F1 |
| 247 | P247 | -0.054 | 0.298 | **0.848** | -0.139 | F3 |
| 248 | P248 | **0.914** | -0.074 | 0.236 | 0.118 | F1 |
| 249 | P249 | **0.939** | 0.216 | 0.048 | -0.057 | F1 |
| 250 | P250 | **0.882** | 0.166 | -0.021 | -0.034 | F1 |
| 251 | P251 | 0.249 | **0.877** | 0.186 | -0.222 | F2 |
| 252 | P252 | 0.149 | 0.662 | 0.675 | -0.126 | - |
| 253 | P253 | 0.295 | **0.719** | 0.430 | -0.331 | F2 |
| 254 | P254 | 0.216 | **0.833** | 0.389 | -0.205 | F2 |
| 255 | P255 | **0.868** | 0.189 | -0.065 | -0.235 | F1 |
| 256 | P256 | 0.149 | 0.346 | **0.647** | -0.514 | F3 |
| 257 | P257 | 0.347 | **0.785** | 0.171 | -0.284 | F2 |
| 258 | P258 | 0.369 | **0.857** | -0.029 | -0.136 | F2 |
| 259 | P259 | 0.110 | **0.866** | 0.346 | -0.178 | F2 |
| 260 | P260 | -0.050 | **0.942** | -0.060 | 0.196 | F2 |
| 261 | P261 | 0.044 | **0.924** | -0.048 | 0.068 | F2 |
| 262 | P262 | -0.072 | 0.159 | **0.772** | 0.400 | F3 |
| 263 | P263 | **0.664** | -0.234 | 0.094 | 0.535 | F1 |
| 264 | P264 | **0.681** | 0.505 | 0.271 | 0.013 | F1 |
| 265 | P265 | 0.170 | **0.774** | 0.327 | -0.405 | F2 |
| 266 | P266 | **0.888** | 0.160 | -0.075 | 0.073 | F1 |
| 267 | P267 | 0.162 | **0.748** | 0.245 | -0.467 | F2 |
| 268 | P268 | **0.907** | -0.143 | 0.038 | 0.017 | F1 |
| 269 | P269 | -0.140 | 0.594 | **0.640** | -0.162 | F3 |
| 270 | P270 | 0.384 | **0.764** | 0.008 | 0.083 | F2 |
| 271 | P271 | **0.876** | 0.342 | -0.180 | -0.001 | F1 |
| 272 | P272 | **0.727** | 0.494 | -0.289 | -0.188 | F1 |
| 273 | P273 | **0.691** | 0.518 | -0.281 | -0.232 | F1 |
| 274 | P274 | **0.828** | 0.359 | -0.129 | -0.149 | F1 |
| 275 | P275 | 0.165 | **0.843** | 0.282 | 0.006 | F2 |
| 276 | P276 | **0.890** | 0.213 | -0.111 | 0.021 | F1 |
| 277 | P277 | **0.761** | -0.058 | 0.470 | 0.294 | F1 |
| 278 | P278 | 0.341 | **0.799** | -0.157 | 0.335 | F2 |
| 279 | P279 | 0.097 | **0.918** | -0.234 | 0.102 | F2 |
| 280 | P280 | **0.858** | 0.100 | -0.272 | -0.108 | F1 |
| 281 | P281 | 0.473 | **0.799** | -0.223 | 0.065 | F2 |
| 282 | P282 | **0.856** | -0.133 | 0.149 | 0.145 | F1 |
| 283 | P283 | 0.000 | **0.884** | 0.341 | -0.011 | F2 |
| 284 | P284 | 0.171 | 0.458 | **0.756** | -0.254 | F3 |
| 285 | P285 | **0.911** | 0.206 | -0.159 | -0.029 | F1 |
| 286 | P286 | **0.937** | 0.137 | 0.178 | 0.103 | F1 |
| 287 | P287 | 0.266 | **0.889** | 0.127 | -0.154 | F2 |
| 288 | P288 | **0.779** | 0.443 | -0.139 | -0.170 | F1 |
| 289 | P289 | **0.727** | 0.081 | -0.132 | 0.211 | F1 |
| 290 | P290 | -0.077 | **0.831** | 0.479 | -0.023 | F2 |
| 291 | P291 | -0.133 | **0.941** | 0.165 | -0.055 | F2 |
| 292 | P292 | **0.805** | 0.172 | 0.319 | 0.219 | F1 |
| 293 | P293 | **0.946** | 0.164 | 0.119 | -0.026 | F1 |
| 294 | P294 | 0.159 | **0.913** | 0.141 | 0.093 | F2 |
| 295 | P295 | 0.141 | **0.845** | 0.312 | 0.200 | F2 |
| 296 | P296 | **0.889** | -0.152 | -0.081 | 0.025 | F1 |
| 297 | P297 | **0.860** | 0.078 | 0.130 | -0.007 | F1 |
| 298 | P298 | 0.446 | **0.821** | 0.065 | -0.053 | F2 |
| 299 | P299 | **0.880** | 0.269 | -0.062 | 0.064 | F1 |
| 300 | P300 | **0.876** | 0.090 | 0.131 | -0.185 | F1 |
| 301 | P301 | 0.298 | **0.860** | 0.094 | -0.190 | F2 |
| 302 | P302 | **0.910** | 0.205 | -0.148 | -0.084 | F1 |
| 303 | P303 | 0.333 | **0.805** | 0.130 | -0.295 | F2 |
| 304 | P304 | **0.835** | 0.386 | -0.283 | -0.121 | F1 |
| 305 | P305 | 0.043 | **0.868** | 0.337 | -0.214 | F2 |
| 306 | P306 | **0.857** | 0.295 | -0.209 | -0.181 | F1 |
| 307 | P307 | 0.145 | **0.931** | 0.045 | -0.107 | F2 |
| 308 | P308 | **0.895** | 0.155 | -0.216 | -0.155 | F1 |
| 309 | P309 | -0.102 | **0.831** | 0.395 | 0.093 | F2 |
| 310 | P310 | **0.819** | 0.348 | -0.205 | -0.070 | F1 |
| 311 | P311 | **0.718** | 0.099 | 0.419 | 0.311 | F1 |
| 312 | P312 | **0.933** | 0.001 | -0.095 | -0.133 | F1 |
| 313 | P313 | **0.951** | 0.153 | -0.053 | -0.034 | F1 |
| 314 | P314 | **0.798** | 0.520 | -0.059 | -0.043 | F1 |
| 315 | P315 | -0.256 | **0.815** | 0.395 | -0.050 | F2 |
| 316 | P316 | 0.137 | **0.851** | 0.366 | 0.031 | F2 |
| 317 | P317 | 0.570 | 0.000 | 0.303 | **0.651** | F4 |
| 318 | P318 | **0.633** | 0.511 | -0.020 | 0.270 | F1 |
| 319 | P319 | 0.642 | **0.676** | 0.088 | 0.074 | F2 |
| 320 | P320 | 0.662 | 0.547 | -0.390 | -0.054 | - |
| 321 | P321 | -0.004 | **0.782** | 0.265 | 0.086 | F2 |
| 322 | P322 | **0.932** | 0.159 | 0.063 | 0.160 | F1 |
| 323 | P323 | -0.285 | **0.691** | 0.278 | 0.477 | F2 |
| 324 | P324 | 0.569 | **0.684** | -0.110 | 0.137 | F2 |
| 325 | P325 | **0.832** | 0.398 | -0.177 | -0.106 | F1 |
| 326 | P326 | 0.630 | 0.672 | 0.050 | -0.245 | - |
| 327 | P327 | 0.150 | **0.931** | 0.122 | -0.088 | F2 |
| 328 | P328 | 0.459 | **0.751** | 0.006 | 0.361 | F2 |
| 329 | P329 | 0.538 | 0.060 | 0.540 | 0.566 | - |
| 330 | P330 | 0.578 | -0.139 | 0.144 | **0.693** | F4 |
| 331 | P331 | 0.172 | **0.930** | 0.103 | 0.033 | F2 |
| 332 | P332 | **0.796** | 0.240 | 0.306 | 0.135 | F1 |
| 333 | P333 | -0.034 | **0.891** | 0.140 | 0.231 | F2 |
| 334 | P334 | 0.516 | -0.242 | 0.031 | **0.629** | F4 |
| 335 | P335 | -0.002 | **0.948** | -0.143 | 0.010 | F2 |
| 336 | P336 | 0.311 | **0.863** | -0.041 | 0.171 | F2 |
| 337 | P337 | -0.091 | **0.680** | 0.599 | -0.169 | F2 |
| 338 | P338 | 0.354 | **0.699** | 0.440 | 0.046 | F2 |
| 339 | P339 | **0.892** | 0.025 | 0.092 | 0.269 | F1 |
| 340 | P340 | 0.029 | **0.838** | 0.203 | 0.414 | F2 |
| 341 | P341 | -0.065 | **0.929** | -0.021 | 0.032 | F2 |
| 342 | P342 | **0.819** | -0.191 | -0.161 | 0.195 | F1 |
| 343 | P343 | **0.570** | -0.229 | 0.044 | 0.425 | F1 |
| 344 | P344 | -0.089 | 0.582 | **0.708** | -0.021 | F3 |
| 345 | P345 | -0.063 | **0.762** | 0.318 | 0.444 | F2 |
| 346 | P346 | **0.931** | 0.182 | 0.063 | 0.100 | F1 |
| 347 | P347 | 0.546 | **0.772** | -0.119 | 0.063 | F2 |
| 348 | P348 | 0.035 | 0.350 | **0.807** | 0.011 | F3 |
| 349 | P349 | **0.878** | -0.032 | 0.166 | 0.152 | F1 |
| 350 | P350 | 0.346 | **0.860** | -0.194 | -0.007 | F2 |
| 351 | P351 | **0.933** | 0.162 | 0.036 | 0.125 | F1 |
| 352 | P352 | 0.529 | 0.532 | -0.100 | -0.254 | - |
| 353 | P353 | **0.808** | 0.469 | -0.018 | 0.116 | F1 |
| 354 | P354 | **0.826** | 0.350 | -0.065 | 0.067 | F1 |
| 355 | P355 | **0.939** | 0.027 | 0.029 | 0.161 | F1 |
| 356 | P356 | **0.829** | -0.032 | 0.317 | 0.398 | F1 |
| 357 | P357 | 0.468 | **0.715** | -0.191 | -0.256 | F2 |
| 358 | P358 | **0.902** | 0.137 | -0.146 | -0.069 | F1 |
| 359 | P359 | 0.443 | **0.563** | 0.131 | 0.088 | F2 |
| 360 | P360 | 0.226 | **0.863** | -0.221 | -0.222 | F2 |
| 361 | P361 | **0.873** | 0.317 | -0.099 | 0.135 | F1 |
| 362 | P362 | -0.139 | 0.568 | **0.669** | 0.155 | F3 |
| 363 | P363 | **0.863** | 0.283 | -0.135 | -0.062 | F1 |
| 364 | P364 | 0.436 | **0.777** | -0.292 | 0.034 | F2 |
| 365 | P365 | 0.305 | **0.797** | 0.051 | 0.254 | F2 |
| 366 | P366 | **0.935** | 0.224 | 0.024 | 0.049 | F1 |
| 367 | P367 | 0.368 | **0.876** | 0.001 | -0.150 | F2 |
| 368 | P368 | -0.131 | **0.853** | 0.135 | -0.048 | F2 |
| 369 | P369 | -0.149 | **0.863** | 0.146 | 0.230 | F2 |
| 370 | P370 | **0.945** | 0.064 | 0.042 | 0.022 | F1 |
| 371 | P371 | -0.126 | **0.925** | 0.034 | 0.053 | F2 |
| 372 | P372 | 0.309 | **0.823** | 0.143 | -0.028 | F2 |
| 373 | P373 | -0.106 | **0.916** | 0.199 | 0.077 | F2 |
| 374 | P374 | -0.089 | **0.878** | 0.261 | 0.151 | F2 |
| 375 | P375 | 0.558 | 0.048 | 0.371 | **0.678** | F4 |
| 376 | P376 | 0.275 | 0.520 | 0.551 | 0.430 | - |
| 377 | P377 | **0.874** | 0.002 | 0.021 | 0.373 | F1 |
| 378 | P378 | 0.409 | **0.590** | -0.069 | 0.393 | F2 |
| 379 | P379 | 0.500 | **0.772** | -0.214 | -0.201 | F2 |
| 380 | P380 | -0.037 | **0.902** | 0.303 | -0.088 | F2 |
| 381 | P381 | 0.007 | **0.799** | 0.535 | 0.063 | F2 |
| 382 | P382 | -0.026 | **0.793** | 0.460 | 0.077 | F2 |
| 383 | P383 | **0.897** | 0.170 | 0.150 | 0.159 | F1 |
| 384 | P384 | 0.397 | **0.820** | -0.130 | 0.030 | F2 |
| 385 | P385 | -0.161 | **0.807** | 0.363 | 0.281 | F2 |
| 386 | P386 | -0.027 | **0.883** | 0.299 | 0.095 | F2 |
| 387 | P387 | -0.258 | **0.857** | 0.294 | 0.028 | F2 |
| 388 | P388 | -0.303 | **0.727** | 0.501 | 0.013 | F2 |
| 389 | P389 | 0.277 | 0.638 | 0.592 | 0.135 | - |
| percent explained variance |  | 39.000 | 30.000 | 13.000 | 5.000 |  |

Table S4. Overview of the defining statements (±4 and 5) and distinguishing statements (at p < 0.05) for factor 2.

| **Num.** | **Statements** | **Factor 2** | **Con/Dist** | **Factor 1** | **Factor 3** | **Factor 4** |
| --- | --- | --- | --- | --- | --- | --- |
| ***S***_47_ | The ground-floor exterior corridor includes openings and equipment access doors, parts of the facade are finished with stone cladding, and the line of sight terminates at an activity node within the courtyard. | 5 | D* | 1 | 1 | 1 |
| ***S***_52_ | An outdoor shaded corridor with a transparent roof, using a metal frame and translucent materials, is provided, with a tiled pedestrian walkway beneath it, flowerbeds and a variety of greenery including trees and shrubs alongside, the environment is orderly, and the corridor provides outdoor circulation and a place to rest. | 5 |  | 3 | 5 | 3 |
| ***S***_31_ | The main exterior facade of the building is in light tones, the decorative style is simple, and the structure is multi-story. | 4 | D* | 0 | 2 | 0 |
| ***S***_33_ | The main facade uses faux-brick finishes and brick-pattern cladding, the decorative style is simple, and columns support an eave corridor. | 4 | D* | -1 | -1 | -1 |
| ***S***_26_ | The floor surface is finished with a wear-resistant coating, and the wall surface features a two-tone protective wall treatment. | 4 | D* | -1 | 2 | 0 |
| ***S***_46_ | At the corner, an arched entrance and a gated wall opening are provided, and the entrance includes fencing and a header sign. | 3 | D* | -2 | -2 | -2 |
| ***S***_48_ | The entrance is equipped with double doors and an accessible ramp in front, and handrails are installed on both sides of the ramp. | 3 | D* | -2 | -2 | 0 |
| ***S***_45_ | The pedestrian path runs parallel to the building facade, it features grid-pattern paving with edge bands, and the width allows two-way circulation. | 3 | D* | -2 | 2 | 1 |
| ***S***_30_ | Display boards and traditional cultural motifs are mounted on the walls, the floor surface is slip-resistant, and lighting is sufficient. | 2 | D* | 2 | -1 | 0 |
| ***S***_29_ | Decorative hanging ornaments are suspended from the ceiling, cultural slogans and wall decorations are displayed on the walls, the color scheme is vivid, and indoor plants are provided. | 2 | D* | 0 | 1 | -2 |
| ***S***_44_ | At the outdoor entrance, the pedestrian walkway and the vehicular lane are separated by a curb, and tactile paving for accessibility is installed along the curb. | 2 | D* | -3 | 0 | -3 |
| ***S***_32_ | The main building facade is primarily light-colored and in a neo-Chinese decorative style, the roof is tiled, and the windows are aligned in an orderly manner. | 2 | D* | -1 | -2 | -3 |
| ***S***_3_ | The exterior facade is tidy, and the fixtures are intact. | 1 | D | -1 | 1 | -2 |
| ***S***_28_ | Decorative artwork and informational signage are affixed to the walls. | 1 | D* | -2 | -5 | -1 |
| ***S***_43_ | A vehicular lane with an asphalt surface is provided on one side of the entrance, which forms a boundary with the adjacent pedestrian walkway. | 0 | D* | -5 | -1 | -5 |
| ***S***_12_ | The bathroom walls are finished with small square tiles, towel racks, shelving, and storage containers for daily items are provided, the layout is compact yet functionally complete, and the circulation space can accommodate wheelchair passage. | 0 | D* | 2 | 1 | 2 |
| ***S***_42_ | Round dining tables with rotating trays and chairs are provided, together with a plant wall and a service counter, and the area is spacious and wheelchair-accessible. | -1 | D* | 3 | 1 | 5 |
| ***S***_39_ | The open spatial layout includes a relatively long corridor with a wide line of sight, the space is broad and supports smooth wheelchair passage. | -1 | D* | 0 | 1 | 3 |
| ***S***_2_ | The environment is properly maintained and clean, the facilities are intact, and the outdoor space can be used for social interaction and rest. | -1 | D | 0 | 3 | 1 |
| ***S***_9_ | The open living area is spacious and wheelchair-accessible, it presents warm tonal contrasts, it is furnished with a television and indoor plants, the layout supports social interaction and rest, and the environment is orderly. | -1 | D* | 2 | 3 | 2 |
| ***S***_40_ | Sets of tables and chairs are arranged in the common area, forming a social seating layout. | -2 | D* | 1 | 0 | 2 |
| ***S***_38_ | A relatively independent interior functional area has a wide visual field, the space is generous and wheelchair-accessible. | -2 | D* | 1 | 0 | 3 |
| ***S***_7_ | The layout is open with a wide visual field, it is furnished with mahjong tables, seating, and sofas, the space is spacious and wheelchair-accessible, and it is suitable for group leisure and recreation. | -2 | D* | 2 | 2 | 3 |
| ***S***_50_ | The indoor rehabilitation area is equipped with treadmills, parallel bars, steps and ramps, and stationary cycling equipment, and activity subzones are designated. | -3 | D* | -2 | 0 | 4 |
| ***S***_37_ | The space is wide, and wheelchair circulation is possible. | -3 | D* | -1 | 0 | 2 |
| ***S***_16_ | The dedicated functional area is furnished with a nursing bed and a bedside cabinet, bedding is complete, and daily-use items such as a fan are placed beside the bed. | -3 | D* | 0 | -2 | 1 |
| ***S***_34_ | Ventilation is achieved through operable external windows together with air conditioning. | -4 |  | -3 | -3 | -4 |
| ***S***_51_ | The interior is furnished with multiple rehabilitation devices (training carts, parallel bars, rehabilitation chairs) and with tables and chairs. | -4 | D* | 0 | -2 | 5 |
| ***S***_49_ | The layout is open with a wide visual field, multiple sets of rehabilitation and exercise equipment and suspended training devices are provided. | -5 | D* | -3 | -1 | 2 |
| ***S***_35_ | Air conditioning is supplied through ceiling-mounted air diffusers. | -5 |  | -4 | -5 | -4 |
| *Distinguishing statements at p < 0.01. | | | | | | |

**Table S5**. Overview of the defining statements (±4 and 5) and distinguishing statements (at p < 0.05) for factor 3.

| **Num.** | **Statements** | **Factor 3** | **Con/Dist** | **Factor 1** | **Factor 2** | **Factor 4** |
| --- | --- | --- | --- | --- | --- | --- |
| ***S***_53_ | A wooden pavilion with a tiled roof is installed under tree shade, with a stone-paved ground surface below, and surrounding trees, fencing, and steps, the environment is orderly, and the pavilion provides a shaded outdoor place to stop and rest. | 5 | D* | 0 | 2 | 1 |
| ***S***_52_ | An outdoor shaded corridor with a transparent roof, using a metal frame and translucent materials, is provided, with a tiled pedestrian walkway beneath it, flowerbeds and a variety of greenery including trees and shrubs alongside, the environment is orderly, and the corridor provides outdoor circulation and a place to rest. | 5 |  | 3 | 5 | 3 |
| ***S***_15_ | The site includes paved walkways bordered by shrubs and trees on both sides, vegetation coverage is high and healthy, and high-rise buildings are visible in the distance. | 4 | D* | -4 | 1 | -3 |
| ***S***_14_ | The site is surrounded by vegetation, including trees and shrubs, which provide shade and visual screening. | 4 | D* | -1 | 1 | -1 |
| ***S***_54_ | In the outdoor garden, fixed outdoor seating and lighting poles are visible, the path is finished with wood decking, and a transparent guardrail is installed alongside. | 4 | D* | 2 | 3 | 1 |
| ***S***_2_ | The environment is properly maintained and clean, the facilities are intact, and the outdoor space can be used for social interaction and rest. | 3 | D* | 0 | -1 | 1 |
| ***S***_1_ | The environment is well maintained and clean, the facilities are intact and orderly, the building is integrated with greenery, and the outdoor space is open, supporting surrounding activities and circulation. | 3 | D* | -2 | 0 | -3 |
| ***S***_13_ | The outdoor environment features abundant greenery and natural elements, trees and shrubs are well maintained, and vegetation coverage is high and shows healthy growth. | 3 | D* | -5 | -4 | -5 |
| ***S***_3_ | The exterior facade is tidy, and the fixtures are intact. | 1 | D* | -1 | 1 | -2 |
| ***S***_25_ | The ground surface is paved with asphalt, which is even and easy to traverse. | 1 | D* | -4 | -2 | -2 |
| ***S***_21_ | Natural light is brought indoors through doors and windows, ceiling-mounted fixtures provide illumination, and smoke-extraction equipment is installed to ensure ventilation. | -3 | D* | 4 | 0 | 0 |
| ***S***_11_ | The sanitary unit includes a washbasin, a mirrored cabinet, and a toilet, and parts of the wall surface are finished with mosaic tiles. | -3 | D | -1 | -2 | 0 |
| ***S***_22_ | Ceiling-mounted fixtures, recessed downlights, and wall-mounted indirect lighting are provided. | -3 | D* | 2 | 0 | -1 |
| ***S***_19_ | The interior features large windows with venetian blinds, allowing natural light in. | -3 | D* | 4 | -1 | -2 |
| ***S***_36_ | Ventilation is provided by ceiling-mounted exhaust together with operable window sashes. | -4 |  | -3 | -3 | -4 |
| ***S***_17_ | The bedroom is furnished with two single beds with the headboards aligned against the wall, and seating and a small round table are placed by the window. | -4 | D* | 1 | -3 | -1 |
| ***S***_23_ | Linear light strips are installed along the wall line of the ceiling together with air diffusers, and the integrated ceiling design improves lighting uniformity. | -4 | D* | 1 | 0 | -1 |
| ***S***_35_ | Air conditioning is supplied through ceiling-mounted air diffusers. | -5 |  | -4 | -5 | -4 |
| ***S***_28_ | Decorative artwork and informational signage are affixed to the walls. | -5 | D* | -2 | 1 | -1 |
| *Distinguishing statements at p < 0.01. | | | | | | |

**Table S6.** Overview of the defining statements (±4 and 5) and distinguishing statements (at p < 0.05) for factor 4.

| Num. | Statements | Factor 4 | Con/Dist | Factor 1 | Factor 2 | Factor 3 |
| --- | --- | --- | --- | --- | --- | --- |
| ***S***_51_ | The interior is furnished with multiple rehabilitation devices (training carts, parallel bars, rehabilitation chairs) and with tables and chairs. | 5 | D* | 0 | -4 | -2 |
| ***S***_42_ | Round dining tables with rotating trays and chairs are provided, together with a plant wall and a service counter, and the area is spacious and wheelchair-accessible. | 5 |  | 3 | -1 | 1 |
| ***S***_8_ | Display areas are integrated with the corridor space, accessible handrails or wayfinding signage are visible, and the area is open, spacious, and wheelchair-accessible. | 4 | D* | 1 | 1 | 0 |
| ***S***_50_ | The indoor rehabilitation area is equipped with treadmills, parallel bars, steps and ramps, and stationary cycling equipment, and activity subzones are designated. | 4 | D* | -2 | -3 | 0 |
| ***S***_7_ | The layout is open with a wide visual field, it is furnished with mahjong tables, seating, and sofas, the space is spacious and wheelchair-accessible, and it is suitable for group leisure and recreation. | 3 | D | 2 | -2 | 2 |
| ***S***_38_ | A relatively independent interior functional area has a wide visual field, the space is generous and wheelchair-accessible. | 3 | D* | 1 | -2 | 0 |
| ***S***_39_ | The open spatial layout includes a relatively long corridor with a wide line of sight, the space is broad and supports smooth wheelchair passage. | 3 | D* | 0 | -1 | 1 |
| ***S***_37_ | The space is wide, and wheelchair circulation is possible. | 2 | D* | -1 | -3 | 0 |
| ***S***_49_ | The layout is open with a wide visual field, multiple sets of rehabilitation and exercise equipment and suspended training devices are provided. | 2 | D* | -3 | -5 | -1 |
| ***S***_12_ | The bathroom walls are finished with small square tiles, towel racks, shelving, and storage containers for daily items are provided, the layout is compact yet functionally complete, and the circulation space can accommodate wheelchair passage. | 2 | D | 2 | 0 | 1 |
| ***S***_29_ | Decorative hanging ornaments are suspended from the ceiling, cultural slogans and wall decorations are displayed on the walls, the color scheme is vivid, and indoor plants are provided. | -2 | D* | 0 | 2 | 1 |
| ***S***_32_ | The main building facade is primarily light-colored and in a neo-Chinese decorative style, the roof is tiled, and the windows are aligned in an orderly manner. | -3 | D | -1 | 2 | -2 |
| ***S***_36_ | Ventilation is provided by ceiling-mounted exhaust together with operable window sashes. | -4 |  | -3 | -3 | -4 |
| ***S***_34_ | Ventilation is achieved through operable external windows together with air conditioning. | -4 |  | -3 | -4 | -3 |
| ***S***_43_ | A vehicular lane with an asphalt surface is provided on one side of the entrance, which forms a boundary with the adjacent pedestrian walkway. | -5 |  | -5 | 0 | -1 |
| ***S***_13_ | The outdoor environment features abundant greenery and natural elements, trees and shrubs are well maintained, and vegetation coverage is high and shows healthy growth. | -5 |  | -5 | -4 | 3 |
| ^⁎^Distinguishing statements at p < 0.01. | | | | | | |

**Text S1:** Audit framework for the built environment of nursing homes oriented toward chronic-care and health-supportive rehabilitation for older adults.

You are an audit assistant for the built environment. Your task is to conduct an academic, systematic audit of environmental features in images of eldercare facilities, rather than offering subjective judgments.

**Workflow**

Identify image type: classify each image as interior or exterior.

Match dimensions: if interior, use only the interior dimensions; if exterior, use only the exterior dimensions.

Objective audit: describe strictly according to the listed dimensions; do not describe elements or dimensions that are not visible.

Free dimension: if important features fall outside the predefined dimensions, add them under the free-description dimension.

**Interior dimensions**

1. Daylighting and lighting conditions (window orientation and area; number and color/temperature of luminaires; blackout/shading curtains).
2. Ventilation and indoor air quality (e.g., operable doors/windows; mechanical ventilation/air-purification devices).
3. Spatial layout and privacy (open-plan vs. compartmentalized functional zones; corridor length; extent of visual exposure/sightlines).
4. Safety and assistive features (e.g., bathroom grab bars; corridor lighting; handrail–wall luminance contrast; wayfinding signage; nurse-call buttons).
5. Spatial capacity (e.g., overall spaciousness; wheelchair accessibility and maneuvering clearance).
6. Comfortable and flexible furniture (e.g., relative placement of beds to windows/desks; social seating configurations).
7. Environmental aesthetics and art elements (e.g., decorative objects and style; indoor plants; proportion of timber; material reflectance; color palette and saturation).

**Exterior dimensions**

1. Greening and natural elements (e.g., vegetation coverage; species richness and health; water features).
2. Accessible Design and Facilities (e.g., entrances and site boundaries; fencing; handrails; sidewalk/path width; outdoor furniture; color contrast).
3. Main-building design (e.g., façade articulation/complexity; architectural style; materials; color scheme; visual interest).
4. Entrance forecourt and seating (e.g., provision of seating in the entrance plaza; steps; landscape ornaments; scale/proportions).
5. Paths and Surface conditions (e.g., walkway width; paving materials; diversity of route types).
6. Shade and shelter (e.g., shaded/rain pavilions; terraces; overhead canopies).
7. Visibility and night-time lighting (e.g., pedestrian-path and façade lighting provision).
8. Proximity and activity intensity (e.g., connectivity to commercial streets; openness; streetscape vibrancy; access to public services).
9. Environmental quality (e.g., cleanliness and maintenance; condition of facilities; orderliness).
10. Social opportunities (e.g., activity/interaction amenities; furniture for gathering; family-visit and group-activity facilities).

**Free dimension**

Identify and add any salient environmental attributes not covered above—for example, cultural symbols, pet-friendly amenities, digital devices/displays, or specialized activity rooms.

**Output requirements**

1. Produce one detailed paragraph per image (~200 words, expandable as needed).
2. Do not describe elements or dimensions that are not visible.
3. Keep descriptions objective and neutral, emphasizing observable characteristics such as quantity, location, materials, scale, and layout.
4. Consolidate outputs into a single text without image numbers or filenames.
5. Each image’s description forms an independent paragraph, separated by one blank line.

**Examples**

Input (interior dining room): large windows + blackout curtains; a table with four chairs; paintings on the wall; an emergency call button.
Output:
The interior features large windows equipped with blackout curtains. A dining table is accompanied by four chairs. Artwork is mounted on the wall, and an emergency call button is installed near the entrance.

Input (exterior garden): lawn coverage; three wooden benches; path lighting; a small fountain in the corner.
Output:
The lawn exhibits high coverage, with three wooden benches provided. Lighting posts line both sides of the footpath, and a small ornamental fountain is placed in the corner.
